# Integrative interactomics applied to bovine fescue toxicosis

**DOI:** 10.1038/s41598-022-08540-2

**Published:** 2022-03-22

**Authors:** Ryan S. Mote, Nicholas S. Hill, Joseph H. Skarlupka, Jessica M. Carpenter, Jeferson M. Lourenco, Todd R. Callaway, ViLinh T. Tran, Ken Liu, Mathew R. Smith, Dean P. Jones, Garret Suen, Nikolay M. Filipov

**Affiliations:** 1 Interdisciplinary Toxicology Program, University of Georgia, 501 D.W. Brooks Dr., Athens, GA 30602 USA; 2 Department of Physiology and Pharmacology, University of Georgia, Athens, GA USA; 3 Department of Crop and Soil Sciences, University of Georgia, Athens, GA USA; 4 Department of Bacteriology, University of Wisconsin – Madison, Madison, WI USA; 5 Department of Animal and Dairy Science, University of Georgia, Athens, GA USA; 6 Division of Pulmonary, Allergy, and Critical Care Medicine, Department of Medicine, Emory University, Atlanta, GA USA

**Keywords:** Computational biology and bioinformatics, Microbiology, Physiology, Systems biology, Biomarkers

## Abstract

Bovine fescue toxicosis (FT) is caused by grazing ergot alkaloid-producing endophyte (*Epichloë coenophiala*)-infected tall fescue. Endophyte’s effects on the animal’s microbiota and metabolism were investigated recently, but its effects *in planta* or on the plant–animal interactions have not been considered. We examined multi-compartment microbiota–metabolome perturbations using multi-‘omics (16S and ITS2 sequencing, plus untargeted metabolomics) in Angus steers grazing non-toxic (Max-Q) or toxic (E+) tall fescue for 28 days and in E+ plants. E+ altered the plant/animal microbiota, decreasing most ruminal fungi, with mixed effects on rumen bacteria and fecal microbiota. Metabolic perturbations occurred in all matrices, with some plant-animal overlap (e.g., Vitamin B6 metabolism). Integrative interactomics revealed unique E+ network constituents. Only E+ had ruminal solids OTUs within the network and fecal fungal OTUs in E+ had unique taxa (e.g., *Anaeromyces*). Three E+-unique urinary metabolites that could be potential biomarkers of FT and targeted therapeutically were identified.

## Introduction

Fescue toxicosis (FT) is a complex livestock disease that occurs when animals graze tall fescue, *Lolium arundinaceum*, infected with the endophyte *Epichloë coenophiala*^1,2^. While the plant-endophyte relationship is considered mutualistic because of beneficial agronomic attributes^3^, *E. coenophiala* produces ergot alkaloids implicated in FT etiology. Thus, wild-type tall fescue is referred to as toxic and leads to production deficits i.e., decreased weight gains^1^, resulting in over $1 billion in annual losses to the US beef industry^4^. Ergot alkaloids are promiscuous, interact with multiple monoamine receptors^5–7^, and elicit systemic pathophysiological responses. In the rumen, ergopeptine alkaloids (e.g., ergovaline) are metabolized to simpler metabolites, i.e., lysergic acid, that are able to cross gastric barriers^8^. This metabolism is likely driven by ruminal hyper-ammonia producing (HAB) and tryptophan-utilizing bacteria^9^, suggesting parent ergopeptine alkaloids may play a limited direct role in the systemic perturbations associated with FT. Considering this, the manner in which other plant-associated molecules and microbiota directly or indirectly affect the toxic (E+) fescue grazing animal, is of interest.

Evidence linking the bovine microbiota and animal productivity has spurred interest in microbiota’s contribution to FT. Altered bacterial ruminal liquid abundances of *Ruminococcaceae*, *Coriobacteriaceae*, and *Erysipelotrichaceae* were reported^10^). Also, decreased diversity and richness were associated with a lower tolerance to FT, while fecal *Neocallimastigaceae* was increased in high-tolerant steers^11^. Additionally, we found that: (i) E+ fescue grazing significantly perturbed certain fecal bacterial taxa, leading to a unique hindgut microbiota structure^12^; (ii) E+ exposure altered the bovine plasma and urine metabolomes^13^; and (iii) plasma and urinary biomarkers of a FT-specific hindgut microbiota were associated with decreased animal performance irrespective of additional external stressors e.g., hot and humid environmental conditions^14^. While these studies were performed using the hindgut microbiota, the foregut (rumen) microbiota is known to contain distinct populations^15^, with both solid and liquid fractions including multiple metabolically important microorganisms^16,17^. Although not evaluated in tall fescue, it is known the phyllosphere microbiota are of concern in grazing diseases like FT, and, besides grazing pressure, respond to factors in the soil, environment and other parasites^18–22^.

No study has yet evaluated microbial and metabolic changes induced in the tall fescue plant by the toxic endophyte, the plant and animal metabolomes/microbiomes simultaneously, or the microbiota–metabolome relationship in multiple biological matrices. To move beyond host–microbe interactions, we applied an integrative analysis of the plant and animal microbiome–metabolome networks as this is expected to provide deeper molecular insights into the FT integrome and help identify molecules, pathways and networks that are directly and/or indirectly responsible for FT and can be targeted therapeutically^23^.

Towards this end, using grazing beef steers, the specific goals of this study were to: (i) characterize the bacterial and fungal microbiota and metabolic differences between non-toxic (Max-Q) and toxic (E+) endophyte-infected tall fescue plant, (ii) assess changes in rumen solid/liquid and fecal microbial (bacterial and fungal) communities and of the rumen, plasma and urine metabolomes that result from E+ grazing, and, importantly (iii) assemble and evaluate the FT integrome by using systems biology approaches applied to the plant–animal microbiome–metabolome networks.

## Materials and methods

### Animals, pastures, and environmental conditions

All animal handling and sample collection were performed in accordance with all relevant guidelines and regulations. The study was carried out in compliance with the ARRIVE guidelines with experimental protocols approved in advance by the Institutional Animal Care and Use Committee of the University of Georgia. Post-weaning Angus steers (n = 12) were blocked by weight and randomly assigned to non-toxic (weight: 306.6 ± 12.2 kg [${\overline{\text{x}}}$ ± SEM]; Max-Q; Jesup MaxQ with endophyte AR542; 3 paddocks; 2 steers per paddock) or toxic (weight: 312.2 ± 16.0 kg; E+; Jesup with wild-type endophyte; 3 paddocks; 2 steers per paddock) tall fescue pastures, as described previously^13^.

### Sample collection and processing

Individual plant tillers were sampled from 15 random locations throughout the pastures and processed for ergot alkaloid analysis as described in^12^ in accordance with the most current guidelines for collection of leaf and plant tissue from cultivated pasture grasses by the College of Agricultural and Environmental Sciences of the University of Georgia, which are aligned with all relevant international guidelines. Temperature and humidity were recorded as in^12^. Steer body weights were recorded prior to (Pre) and at the end (28 days) of the study. Plasma, fecal, and urine samples were collected Pre (Day 0), 2, 7, 14, and 28 days post pasture placement, similar to^12–14^, at a working facility adjacent to the pastures. Voided urine was collected in clean collection cups via a free catch and plasma was harvested from blood collected via jugular blood draw. Ruminal samples on these dates were obtained with an ororuminal probe as in^15^. Rumen liquids and solids were separated with three layers of sterile cheesecloth into 3 mL (liquids) and ~ 5 g (solids) aliquots, frozen immediately on dry ice, then stored at − 80 °C.

### Urinary ergot alkaloid analysis

Total urinary ergot alkaloid concentrations were determined for all samples as previously described^13,24,25^.

### DNA extraction

Genomic DNA was extracted from all bovine matrices as previously described^12,14^. Tall fescue samples were stomached for 5 min in a sterile stomacher bag with TE extraction buffer. The supernatant was collected and subjected to the same extraction procedures as previously described^12,14^.

### DNA amplification and sequencing

Sequencing of the bacterial 16S rRNA gene was performed as described^12,14^. For fungal sequencing, custom primers targeting the 5.8S-internal transcribed spacer 2 (ITS2) region (F-AGCCTCCGCTTATTGATATGCTTAART, R-AACTTTYRRCAAYGGATCWCT) were used, as in^26^. The primers also contained Illumina-specific sequencing adapters (F-AATGATACGGCGACCACCGAGATCTACAC; R- CAAGCAGAAGACGGCATACGAGAT). The following PCR cycling (40 cycles; 30 ng starting DNA) conditions were used: initial denaturation at 95 °C for 3 min; 39 cycles of 95 °C for 30 s, 58 °C for 30 s, 72 °C for 30 s; with the final extension set at 72 °C for 5 min. Controls and both 16S rRNA and ITS2 PCR products were treated as before and sequenced on an Illumina MiSeq using a v2 sequencing reagent kit (500-cycle)^12,14^.

### NGS sequence processing and bioinformatics analysis

Raw 16S rRNA and ITS2 sequence files were processed using mothur v.1.41.3, as previously described^12,14^. After quality filtering, unique sequences were aligned to the SILVA version 119 reference alignment database^27^ and chimeras were removed using *chimera.uchime* (http://drive5.com/uchime). Bacterial sequences were aligned to the Greengenes database v13.8 (http://greengenes.secondgenome.com)^28^. Singletons were removed and the operational taxonomic units (OTUs) were normalized for sequence depth (i.e., each sample was normalized to the number of sequences in the smallest sample) and abundance filtered prior to statistical analysis. ITS2 sequences were aligned to the UNITE database v04.02.2020^29^. Herein, sequences classified as belonging to the genus *Neotyphodium* in UNITE will be referred to its updated genus nomenclature *Epichloë*^2^.

### High-resolution metabolomics (HRM)

Metabolomics sample processing for urine, plasma, and rumen liquids were performed as previously described for plasma and urine^13^. For tall fescue preparation, approximately 50 mg of plant material was added to vials with 100 μL acetonitrile, sonicated for 10 s and placed on ice for 30 min prior to centrifugation (10 min at 14,000 rpm). All HRM samples were analyzed with a Thermo Scientific linear triple quadrupole (LTQ) Orbitrap Velos with either hydrophilic liquid interaction chromatography (Waters Xbridge BEH Amide 2.5 μm, 2.1 × 100 mm) or C18 chromatography (Higgins Targa C18 5 μm, 2.1 × 100 mm) with 10 min gradient runs, positive ionization, and instrument settings at 120,000 resolving power, 5 min runs, and 10 μL injection. Detection of metabolomics features and QC was performed as previously described^13^. All metabolomics annotation presented herein was generated using xMSannotator^30^ and either the Human Metabolome Database HMDB^31^; or the T3DB toxic exposome database T3DB^32^. Pathway analysis was performed using *mummichog*^33^ and annotated with either the bovine or the thale cress KEGG databases for, respectively, bovine and tall fescue samples.

### Overlapping feature analysis

Sets of overlapping OTUs were determined using OTU tables that included only OTUs with greater than 10 sequences (50% presence) within a matrix and a treatment. For the metabolomics overlap, all features that were present in > 80% of samples within a matrix and a treatment were used. Venn Diagrams were generated using the Bioinformatics and Evolutionary Genomics online processor (http://bioinformatics.psb.ugent.be/webtools/Venn/).

### Targeted network analysis

Normalized bacterial and fungal OTUs detected in > 50% of samples in the E+ plant and rumen, specific to each analysis, were correlated to the *Epichloë* OTU using the Hmisc *R* package^34^; network analysis was performed on the significantly (*P* < 0.05) correlated features using qgraph^35^. The same approach was utilized for the rumen liquids metabolomics targeted network correlated to the putatively annotated ergot alkaloid metabolite ergovaline.

### Integrative interactomics analysis

xMWAS v0.552(^32,36^; https://kuppal.shinyapps.io/xmwas/) was used for the integrative interactomics analyses with the following parameters: dataX = metabolomics; dataY = 16S OTU table; dataZ = ITS2 OTU table; RSD > 1; Maximum number of variables from dataX = 500, from dataY and dataZ = 250; integration methods = sparse partial least squares regression (sPLS) in canonical mode with optimization of sPLS components with 500 [dataX], 250 [dataY], and 250 [dataZ] variables selected by sPLS; the association analysis was set to (|r| > 0.5; *P* < 0.05); centrality analysis used eigenvector centrality; graphical options were default. For the fescue xMWAS, parameters were: maximum number of variables from dataX = 500, from dataY and dataZ = 250, correlation threshold (|*r*| > 0.3), 1000 [dataX], 100 [dataY], and 100 [dataZ] variables selected by sPLS. The rumen analysis included the same variables as the fescue plant analysis, but the correlation threshold was set to (|*r*| > 0.5). The output files were downloaded and differential networks were imported into Cytoscape^37^ for graphical visualization and analysis.

### Targeted animal integrative interactomics analysis

Putative metabolites that were significantly affected by E+ grazing and annotated by mummichog using the *bos taurus* KEGG database^38^ into tryptophan metabolism, tyrosine metabolism, Vitamin B6 metabolism, steroid hormone biosynthesis, and primary bile acid metabolism were used to perform these integrative interactomics analysis by targeting metabolic pathways commonly perturbed by E+^12,13^. sPLS was performed using 290 metabolites and microbiota data from the untargeted analysis for both Max-Q and E+ data sets; differential network analysis were performed to identify metabolic and microbial nodes specific to E+ steers in Cytoscape.

### Statistical analysis of weight gains and urinary ergot alkaloids

Analyses of weight gains and urinary ergot alkaloids was performed using Sigma Plot, v 12.5 (Systat Software, Inc., San Jose, CA; https://systatsoftware.com) using two-way ANOVA (within-subjects design) with Holm-Sidak post-hoc analysis performed where applicable as in^12–14^.

### Transparency statement(s)

All DNA sequences are publicly available in the NCBI Sequence Read Archive and are accessible under BioProject accession number PRJNA817179. HRM feature intensity tables and metadata will be deposited in the Metabolomics Workbench (https://www.metabolomicsworkbench.org). 

## Results

Non-integrative results (physiological, microbiota, metabolomics) are presented and elaborated on in the supplemental files; some important outcomes are highlighted below.

### Physiological results

E+ grazing significantly increased urinary ergot alkaloids and reduced cumulative and daily weight gains (Fig. S1), indicating presence of fescue toxicosis.

### Sequencing results

After quality control measures, 16S sequencing resulted in 4,976,758 high-quality sequences and a total of 4911 operational taxonomic units (OTUs) for all samples. The post-QC ITS2 sequencing resulted in 4,518,088 high-quality sequences that clustered into a total of 3116 OTUs across all samples.

### General influence of E+ on plant and animal bacterial and fungal microbiota profiles

For bacterial alpha diversity, Simpson’s diversity was increased by E+ in fescue plant and decreased by E+ in rumen liquids, with rumen liquids also exhibiting effect of time (Fig. S2). There were main effects of E+ and time for Chao1 richness in rumen liquids (increased in E+), rumen solids (increased in E+ after 14 days), and fecal matter (mixed effects; Table S1; Fig. S2). For ITS2 alpha diversity, there was a main effect of E+ (an increase) in rumen solids (Table S1; Fig. S2). Also, there were time effects in several matrices for both 16S and ITS2 diversity (Table S1; Fig. S2). Permutational analysis of variance (PERMANOVA) on bacterial and fungal microbiota revealed significant main effects of E+ and time for Bray–Curtis and Jaccard indices in all sample matrices, except ITS2 in fescue plant (Table S1). The only significant treatment by time interactions were for plant bacterial and fungal ruminal solids and liquids profiles (Table S1). These data indicate that microbiota of the fescue plant, rumen solids, rumen liquids and feces are all perturbed by E+.

### Identification of *E. coenophiala* in the microbiota

After quality filtering, alignment, and normalization for sequencing depth, one OTU aligned to *Epichloë* remained and was detected only in E+ plant and rumen samples, with the greatest rumen abundance being after 14 days of grazing. This OTU was used for subsequent targeted network analysis in the E+ plant and rumen.

### LEfSe results

#### Fescue plant

Most tall fescue bacteria (Fig. S3A) and fungi (Fig. S3B) that were altered by E+ were plant-specific. The most notable effect was increased *Epichloë* in E+ tall fescue. A full list of the significantly different bacteria and fungi between fescue cultivars tall fescue is in the Fig. S3 legend.

#### Rumen solids and liquids

**Figure 1:**
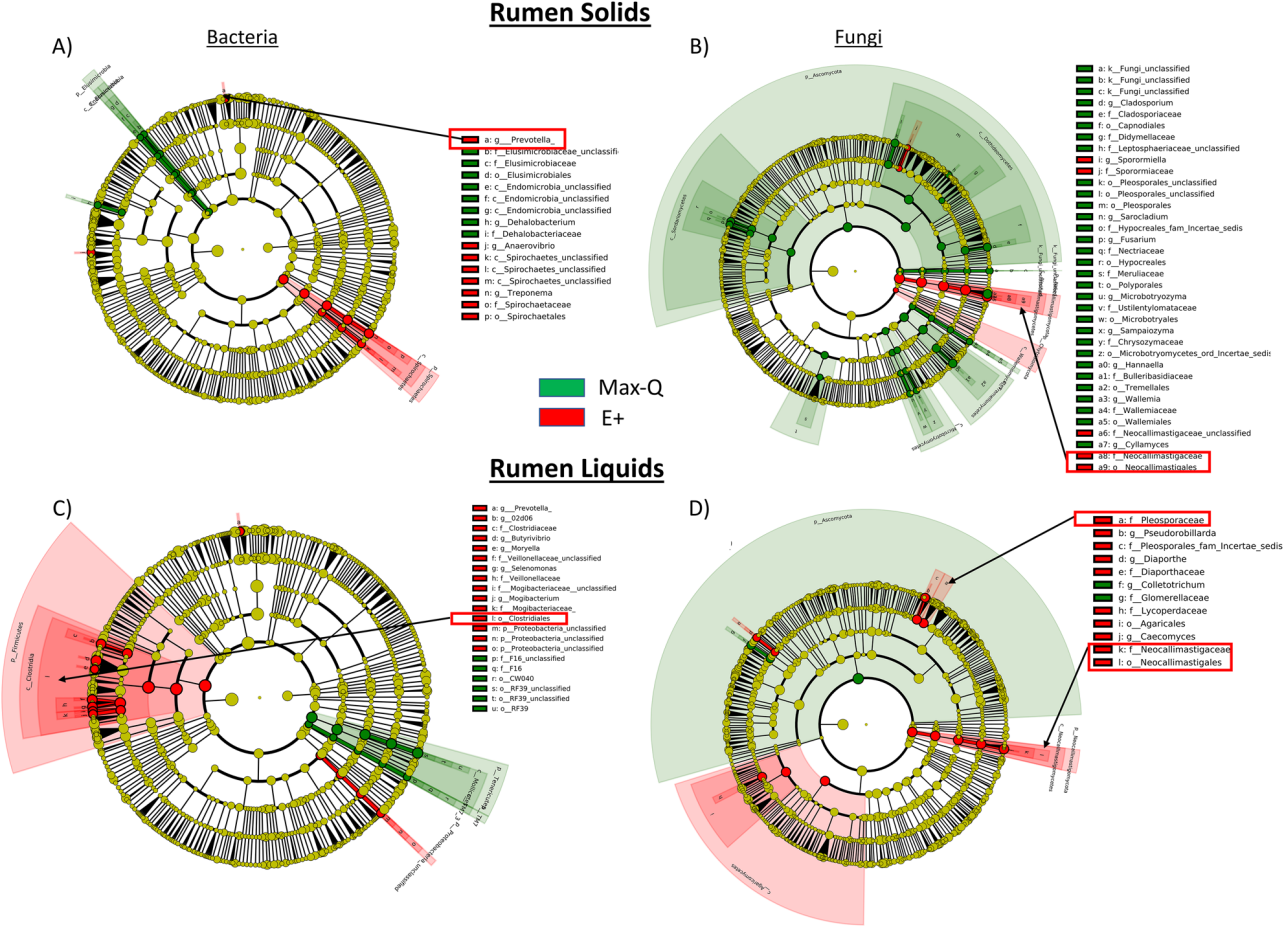
Linear discriminant analysis (LDA) effect size (LEfSe; Kruskal–Wallis [*P* < 0.05]; Pairwise Wilcoxon [*P* < 0.05]; logarithmic LDA score > 2.0) of the rumen solid (**A**) bacterial and (**B**) fungal and rumen liquid (**C**) bacterial and (**D**) fungal microbiota of Angus steers across a 28-day grazing trial after placement on either a non-toxic (Max-Q; n = 6) or toxic (E+; n = 6) endophyte-infected tall fescue. Green and red shading indicates greater abundance in Max-Q or E+ steers, respectively. Taxonomic rank labels are provided before microbe names: “p_; c_; o_; f_; g_” indicate phylum, class, order, family, and genus, respectively. Letters and numbers within the cladograms refer to respective bacterial or fungal names located in the keys to the right of each cladogram. Select taxa of interest are highlighted by boxes and arrows point to their position within a cladogram.

When considering Pre, Max-Q, and E+ steers, the rumen solids and liquids microbiota shifts post-pasture placement (Fig. S4). Focusing on E+ (Fig. 1A), g_*Dehalobacterium*. p_*Spirochaetes* (notably g_*Treponema*), and g_*Prevotella* were all increased. Rumen solids fungi were significantly more abundant for most taxa in Max-Q, but select fungi increased in E+ (Fig. 1B). Examples are g_*Sporormiella,* f_*Neocallimastigaceae*, and p_*Chytridiomycota*. Among E+ effects on bacteria in rumen liquids were increases of g_*Mogibacteriu,* and g_*Prevotella* (Fig. 1C); effects of E+ on rumen liquid fungal communities included increases of f_*Neocallimastigaceae* and f_*Lycoperdaceae* (Fig. 1D). Complete lists of affected bacteria and fungi are located in the legends of Fig. 1.

#### Feces

E+ increased c_*Clostridia,* g_*Mogibacterium*, and g_*Clostridium* (c_*Clostridia*); in the fecal fungal microbiota, it is notable that the c_*Sordariomycetes* and order (o_)*Hypocreales* were increased in E+ steers, but f_*Clavicipitaceae* and g_*Epichloë* were not (Fig. S5). Analysis, including samples prior to pasture placement (Pre), are presented as Fig. S6.

#### Metabolic effects of E+ exposure

**Figure 2:**
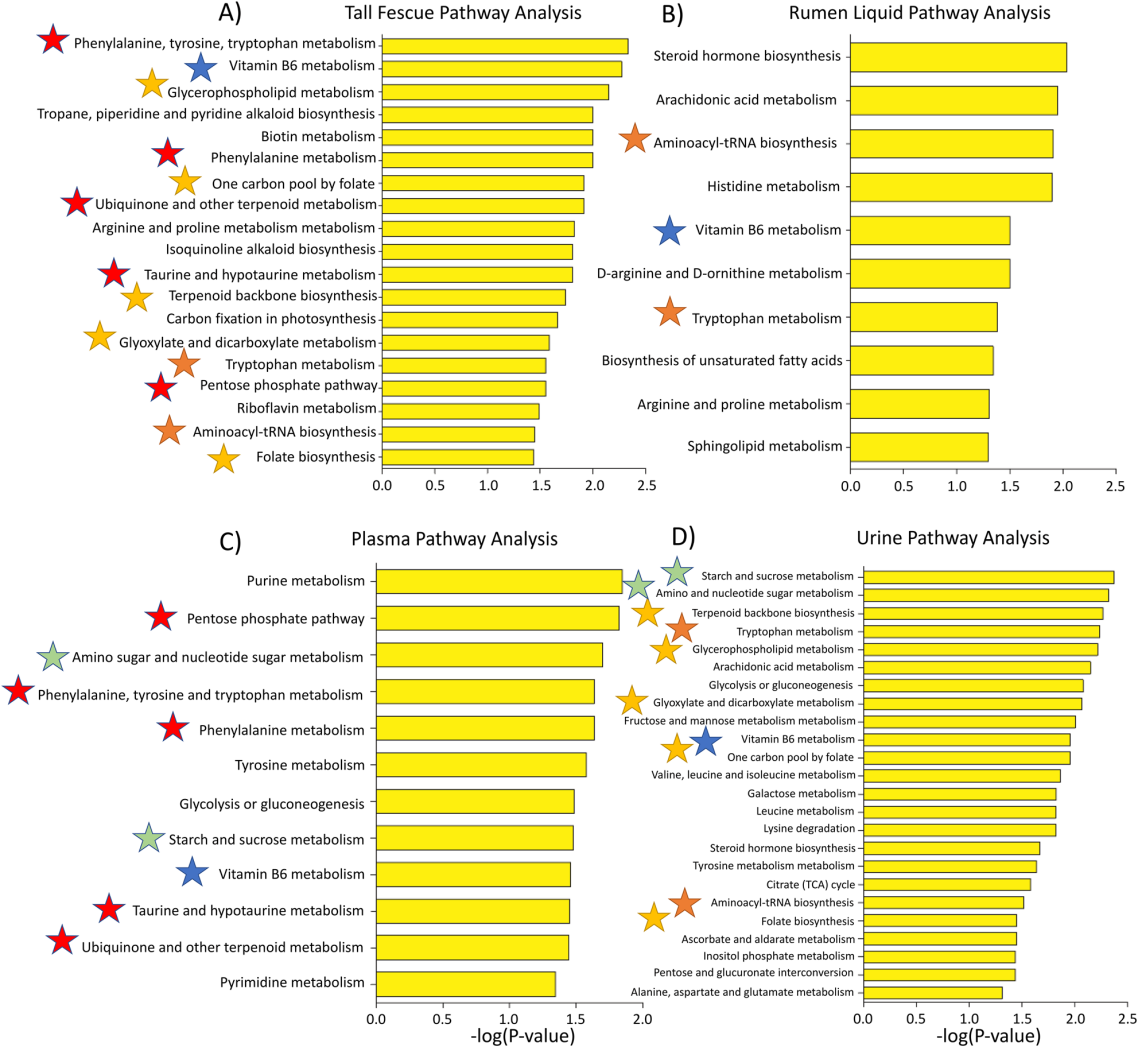
Metabolic pathway analysis performed on the (**A**) tall fescue plant, (**B**) rumen, (**C**) plasma and (**D**) urine high-resolution metabolomics features using mummichog. Putative metabolic pathways significantly (*P* < 0.05) affected by toxic tall fescue (E+) in the plant and animal throughout the 28-day grazing trial are presented. The negative log of the FDR- corrected *P* value for each metabolic pathway indicated on the y-axis is on the x-axis. Blue star signifies overlapping pathways between all biological matrices; orange is fescue grass, rumen liquid, and urine overlap; red is fescue grass and plasma overlap; yellow is fescue grass and urine overlap.

*E. coenophiala*-infection affected numerous metabolic pathways in the fescue plant, including phenylalanine, tyrosine and tryptophan metabolism, Vitamin B6 metabolism, and tropane, piperidine and pyridine alkaloid biosynthesis (Fig. 2A). E+ grazing perturbed steroid hormone biosynthesis, arachidonic acid, histidine, and Vitamin B6 metabolism in the rumen liquids (Fig. 2B); in the plasma, it did so on, among others, pentose phosphate pathway, phenylalanine, tyrosine, tryptophan, and Vitamin B6 metabolism (Fig. 2C). In the urine, E+ effects were on starch and sucrose metabolism, terpenoid backbone biosynthesis, tryptophan and arachidonic acid metabolism (Fig. 2D). Vitamin B6 metabolism was one of few metabolic pathways affected by E+ presence/grazing across all biological matrices; a full list of affected metabolic pathways is in Fig. 2.

#### Putative ergovaline feature intensity

**Figure 3:**
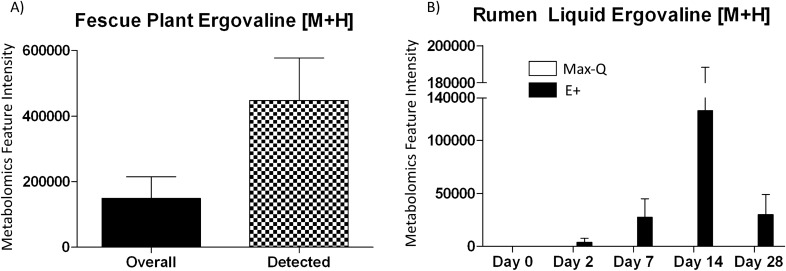
Average putative ergovaline [M + H] feature intensity in (**A**) toxic (E+; n = 18) tall fescue plant for all samples (black) and only in samples where ergovaline was detected (checkered) and (**B**) the rumen liquids of Angus steers grazing either a non-toxic (Max-Q; n = 6) or toxic (E+; n = 6) tall fescue over the course of the 28-day grazing trial. Feature intensity data are presented as mean ± SEM.

Using xMSannotator and the T3DB database, one metabolic feature (*m/z* 534.2708451, time 281.4581094; [M + H]) was putatively identified as ergovaline. Interestingly, this feature was only detected in the E+ plant and rumen liquid. In the E+ fescue, putative ergovaline was detected in multiple individual plant samples, with the average overall and positive sample intensities depicted in Fig. 3A. In the rumen liquid, ergovaline was not detected in any Max-Q or E+ samples before pasture placement; it was first detected in E+ steers on Day 2, peaked on Day 14, with Day 28 levels equaling Day 7 (Fig. 3B).

#### Overlapping microbial and metabolic features

**Figure 4:**
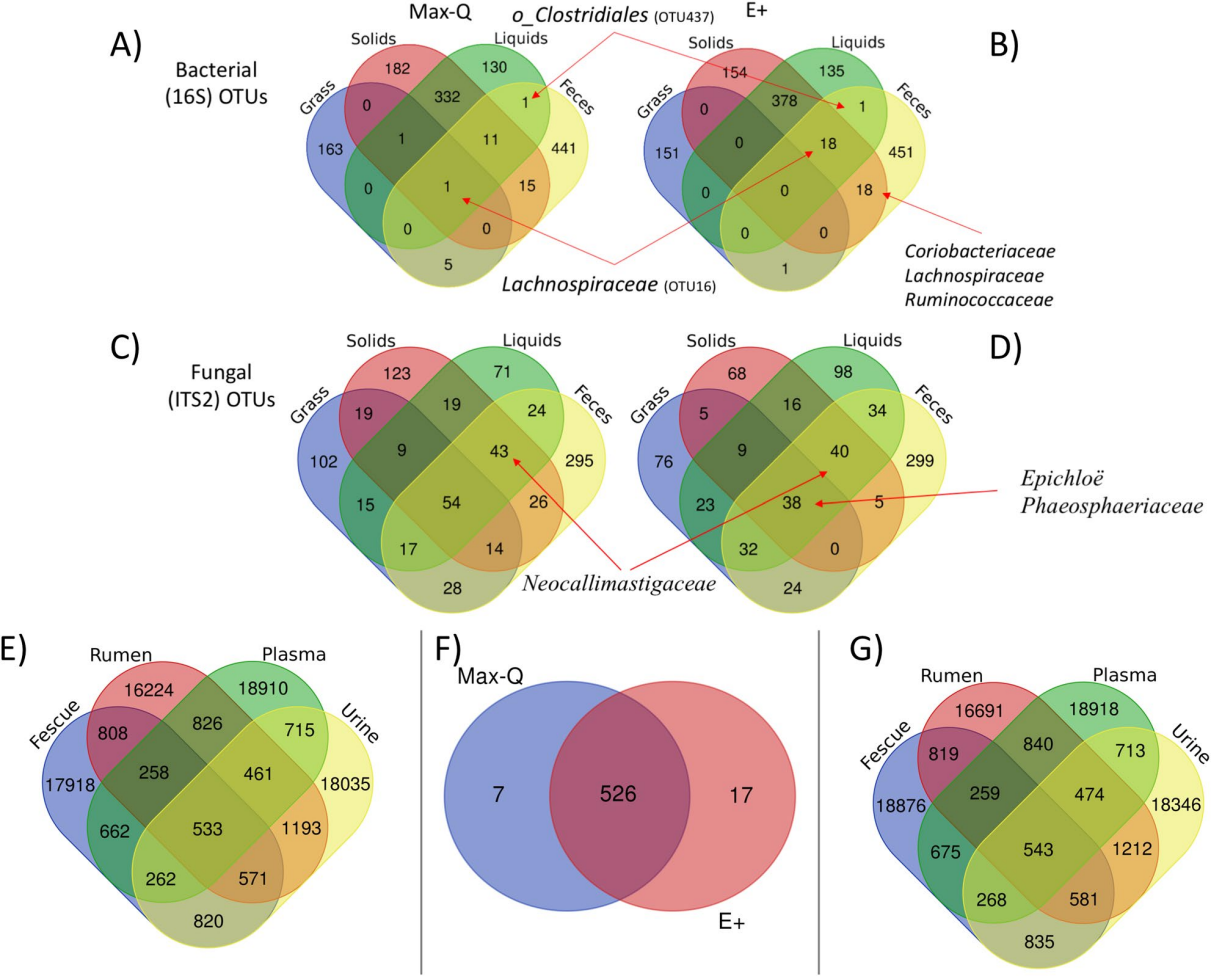
Top, Middle: Venn diagrams representing specific bacterial (16S; **A**, **B**) and fungal (ITS2; **C**, **D**) OTUs that overlapped between biological matrices in steers grazing a novel, non-toxic (Max-Q; n = 6; left) or a toxic (E+; n = 6; right) tall fescue over the course of a 28 day grazing trial. Only OTUs with sequence counts (nseq > 10) were included in the analysis. Red arrows indicate specific microbes of interest with overlapping OTUs. Bottom: Venn diagrams representing specific metabolic features with exact mass-to-charge ratios (*m/z*’s) that overlapped between biological matrices in steers grazing (**E**) a non-toxic (Max-Q; n = 6) or (**G**) a toxic (E+; n = 6) tall fescue over the course of a 28 day grazing trial. (**F**) Represents shared or distinct features between Max-Q and E+ that overlapped between all four biological matrices in each respective cultivar. Only metabolic features present in > 80% of samples within a treatment and matrix were included in the analysis.

Plant and animal bacterial OTUs did not overlap, but an overlap between plant and rumen fungal OTUs (Max-Q 103 OTUs; E+ 115 OTUs) was present. Substantial overlap occurred between bacterial (332 Max-Q; 378 E+) OTUs in the rumen solids and rumen liquids regardless of fescue cultivar (Fig. 4A,B). Overlapping OTUs between the rumen solids and feces within E+ steers mapped to f_*Coriobacteriaceae*, f_*Lachnospiraceae*, and f_*Ruminococcaceae* families (Fig. 4B).

For the fungi, more OTUs overlapped between the grass and rumen liquids than grass and rumen solids in both Max-Q and E+ steers (Fig. 4C,D, respectively)*.* In E+ steers, one OTU aligned to the *Epichloë* genus, alongside multiple *Phaeosphaeriaceae* OTUs, overlapped between these biological matrices. Most OTUs overlapping between the fescue grass, rumen solids, and rumen liquids were cultivar specific, including *Cryptococcus aureus* and *Cryptococcus dimennae* in E+ (Fig. 4D)*.* Multiple f_*Neocallimastigaceae* OTUs overlapped between all animal matrices irrespective of treatment. Sub-family overlap was largely cultivar-specific, and the full list of overlapping bacterial and fungal OTUs is in File S1.

Similar metabolite (*m/z*) overlap was observed for Max-Q and E+ steers. 533 and 543 metabolic features overlapped between all biological matrices in Max-Q (Fig. 4E) and E+ steers (Fig. 4G), respectively. Of these, 526 were shared, and 7 and 17 were distinct to Max-Q and E+, respectively (Fig. 4F). Among the distinct E+ features were metabolites putatively annotated as: 11-dimethoxydecane (*m/z* 102.1042 [M + H]), urea (m/z 121.0718 [2M + H]), L-kynurenine (m/z 209.0921 [M + H]), and (R)-Pterosin B (*m/z* 219.1379), plus several unannotated metabolites.

#### Targeted *Epichloë* and ergovaline correlation network analysis

**Figure 5:**
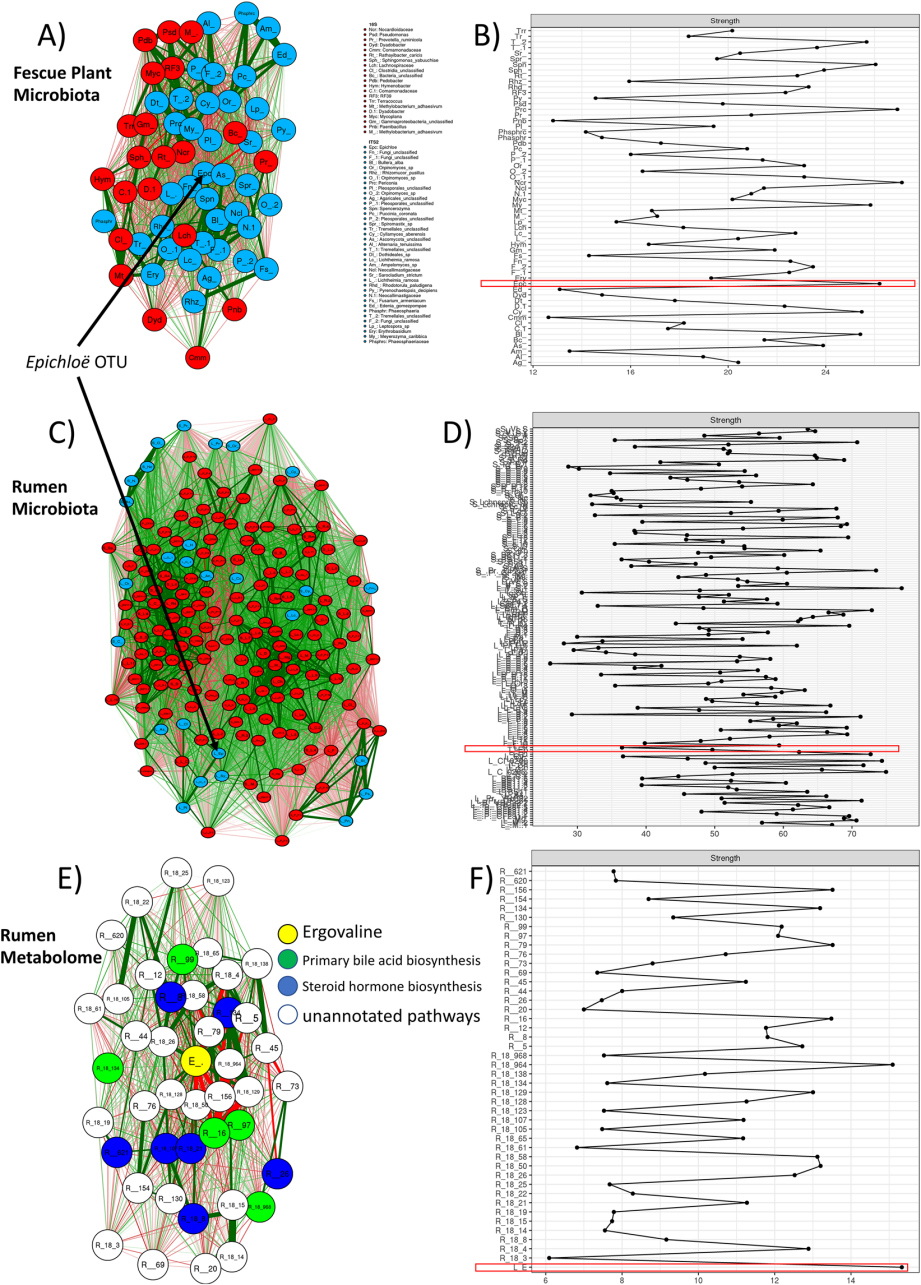
Targeted correlation-based network analysis of significantly correlated features with (**A**, **B**) fescue plant *Epichloë* (*coenophiala)* OTU, (**C**, **D**) rumen liquid *E.(coenophiala)* OTU, and (**E**, **F**) ergovaline. (**A**) Network of toxic tall fescue plant (E+; *P* < 0.05) bacterial and fungal OTUs (**B**) respective centrality measurements with *E. (coenophiala)* marked in red; (**C**) network of toxic tall fescue grazing steers (E+; *P* < 0.05) ruminal bacterial and fungal OTUs with (**D**) respective centrality measurements with *Epichloë* marked in red; (**E**) focused network of toxic tall fescue grazing steers (E+; |r| > 0.6; *P* < 0.05) ruminal metabolic features significantly correlated with ruminal ergovaline and (**F**) respective centrality measurements with (ergovaline*)* marked in red. Blue and red nodes in (**A**, **B**, **C**, **D**) represent fungal and bacterial nodes, respectively. Yellow, green, blue, and white nodes in (**E**, **F**) indicate, respectively, ergovaline, metabolites involved in primary bile acid biosynthesis, metabolites involved in steroid hormone biosynthesis, and metabolites from unannotated pathways. *E. (coenophiala*) presence in the network is highlighted by arrows. Green and red lines indicate positive and negative correlations, respectively.

*E. (coenophiala*) targeted network analysis in the fescue plant revealed one highly interconnected network of 29 fungal and 31 bacterial OTUs (Fig. 5A). Of note, the *E. (coenophiala*) OTU had the third highest centrality measure, preceded only by one *Periconia* and one *Nocardioidaceae* OTU (Fig. 5B). The classification of most OTUs in the network were unique (i.e., 1 OTU per family/genus/species). The only classified taxa with more than 1 OTU were *Lichtheimia ramose*, *Neocallimastigaceae*, *Comamonadaceae*, *Dyadobacter*, and *Methylobacterium adhaesivum*.

For the rumen, targeted analysis was performed using the *E. (coenophiala*) OTU in the rumen liquids; 180 total (27 fungi [8 solids, 19 liquids], 153 bacteria [61 solids, 92 liquids]) OTUs were significantly correlated with the *Epichloë* OTU (Fig. 5C). Of these, 4 liquid and 2 solid *Orpinomyces sp*, 2 solid and 1 liquid *Cyllamyces aberensis*, and 2 *Neocallimastigaceae* OTUs were the most prominent. *Aspergillus cristatus* and *Piromyces sp* were the other fungi in the rumen network that had an OTU from both rumen solids and rumen liquids. From the bacteria, *Prevotella* and *Lachnospiraceae* both had 14 liquid and 13 solid OTUs in the network. Other notable bacteria included *BS11* (7 liquid, 4 solid OTUs), candidate *CF231* (6 liquid OTUs), *Butyrivibrio* (6 liquid, 3 solid OTUs), and *Ruminococcaceae* (5 liquid OTUs). The *Epichloë* OTU had median centrality within the network (Fig. 5D).

Finally, E+ rumen targeted metabolite network using the putative *E. coenophiala*-derived metabolite ergovaline was performed with (|r| > 0.4; *P* < 0.05). 497 metabolic features (255 c18, 242 HILIC) were correlated with the ergovaline feature intensity profile (Fig. S7). Ergovaline-associated metabolites from the rumen liquids were involved in steroid hormone biosynthesis, tryptophan, tyrosine, amino and nucleotide sugar, glucose and energy, and Vitamin B6 metabolism. Considering the large size of this network, the analysis was further restricted (|r| > 0.6; *P* < 0.05). The newly generated network (Fig. 5E) had 43 metabolites, where ergovaline was at the center of the network and had the highest centrality measure (Fig. 5F). Pathway analysis revealed that most metabolites in the focused network were involved in steroid hormone, tryptophan, and tyrosine metabolism.

### Differential integrative interactomics

#### Fescue plant integrative interactomics

**Figure 6:**
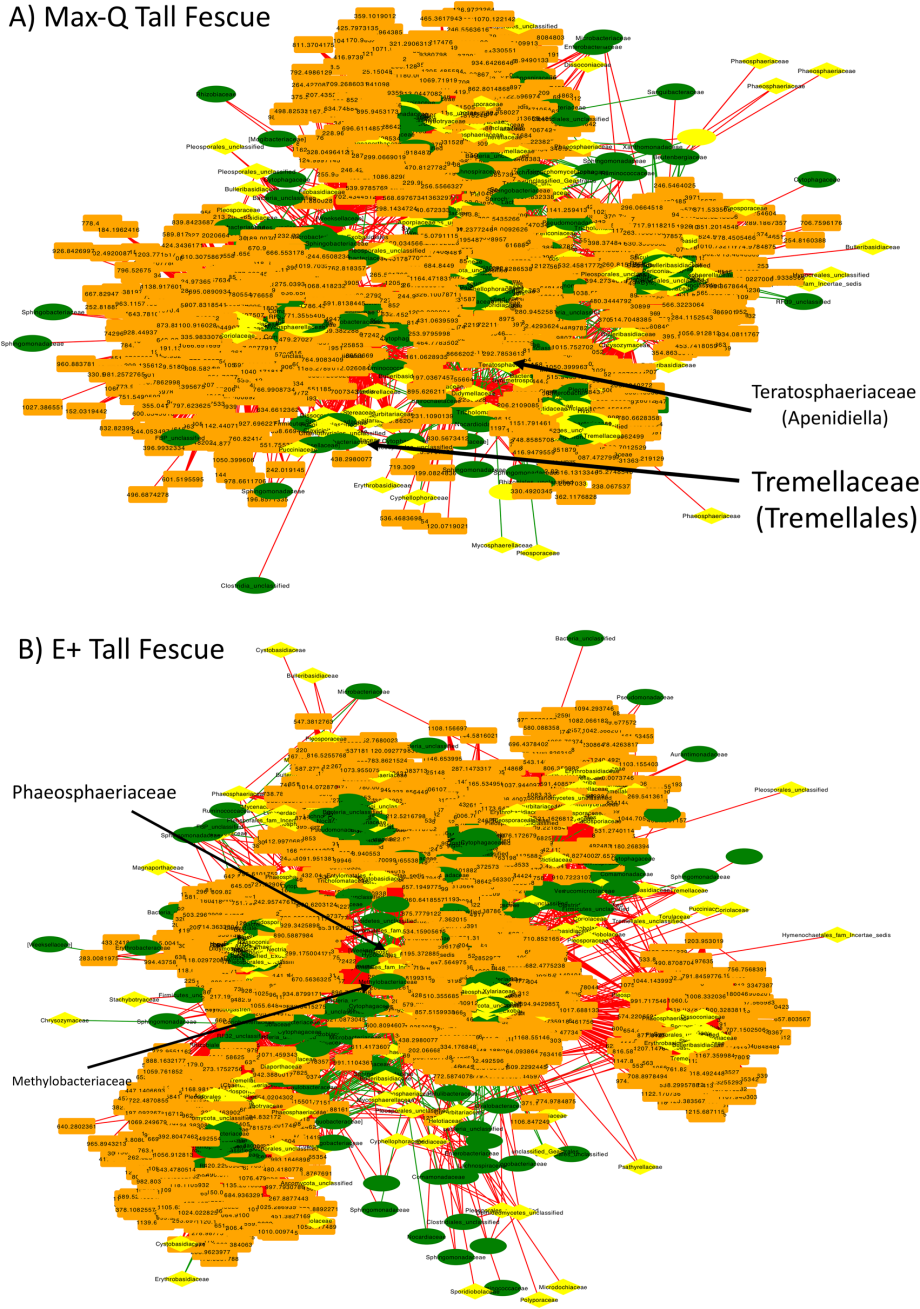
Global fescue plant integrative interactomics networks of relationships between bacterial (green, oval) and fungal (yellow, diamond) OTUs and metabolites (orange, rectangle) in the tall fescue plant within non-toxic (**A**; Max-Q; n = 6) or toxic (**B**; E+; n = 6) endophyte-infected plants. Green and red edges indicate positive and negative correlations, respectively. Select nodes of interest are highlighted by arrows and text.

In both Max-Q and E+ tall fescue networks, bacterial and fungal OTUs were the central nodes in most clusters and were surrounded by associated metabolites (Fig. 6). The Max-Q network consisted of five clusters, whereas the E+ network had seven (Fig. 6). Of the nodes that had centrality measurements > 0.5, 71 were metabolites, 30 were bacteria, and 27 were fungi in the Max-Q network (File S2A); those in the E+ network consisted of 0 metabolites, 22 bacteria, and 34 fungi (File S2B). Cluster 5 of the E+ network (File S2B) had most nodes with high centrality measurements. Notably, in the E+ tall fescue network (Fig. 6; File S2B), no OTU aligning to *E. (coenophiala*), or higher taxonomic levels (e.g., *Clavicipitaceae*), was identified. Numerous OTUs were present only in the E+ tall fescue network, such as *Clostridium* (*Ruminococcaceae*), *Cladosporium*, *and Mogibacteriaceae* (File S2B). Interestingly, the steroid biosynthesis, nucleic acid, and glucosinolate biosynthesis pathways appeared in both Max-Q and E+ networks when querying nodes with centrality measurements above (> 0.2). Metabolic pathways singular to E+ include, among others, ubiquinone and other terpenoid-quinone biosynthesis, tyrosine metabolism, brassinosteroid biosynthesis, and valine, leucine and isoleucine metabolism.

#### Rumen integrative interactomics

**Figure 7:**
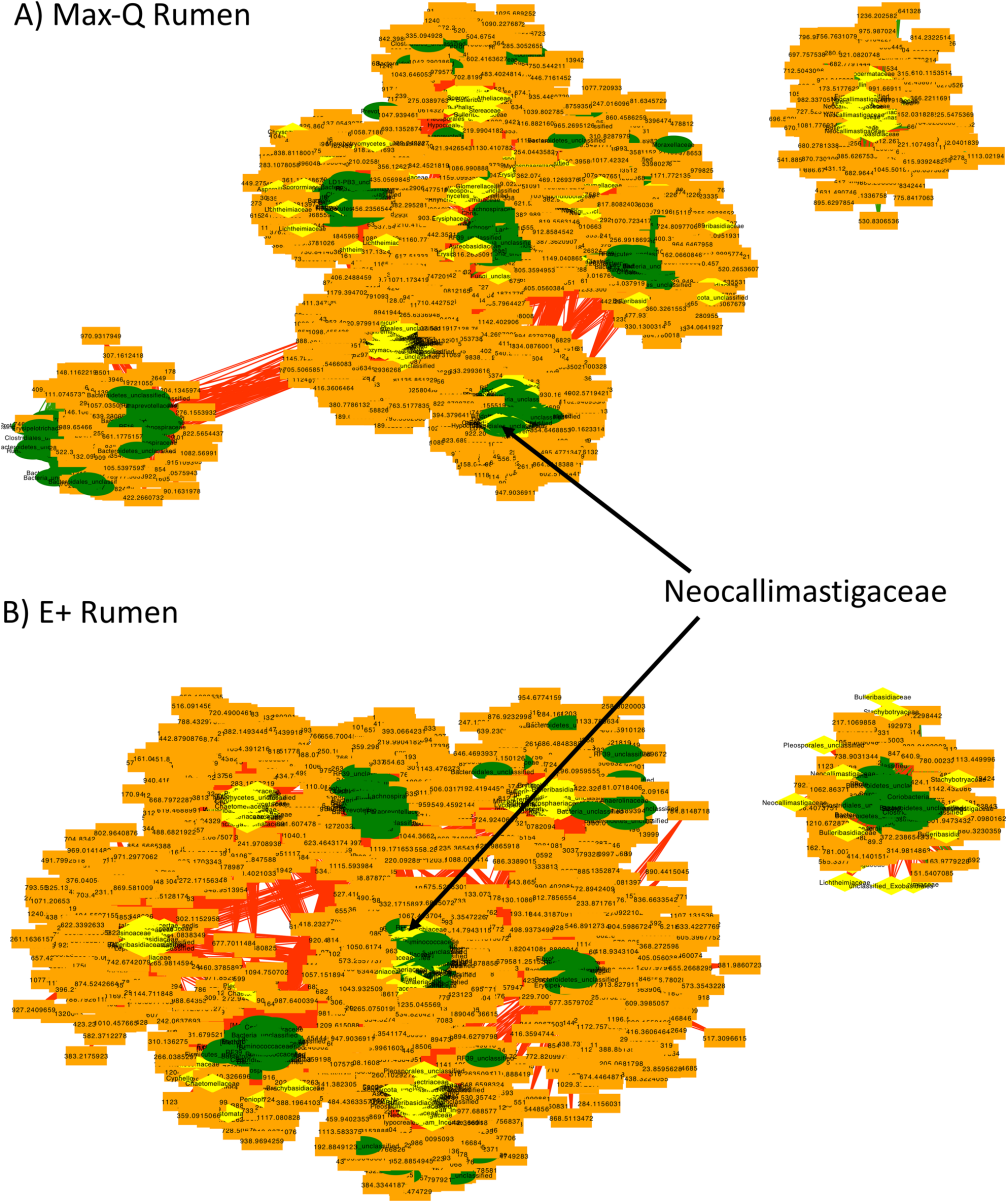
Global rumen integrative interactomics networks demonstrating relationships between bacterial (green, oval) and fungal (yellow, diamond) OTUs and metabolites (orange, rectangle) of non-toxic (**A**; Max-Q; n = 6) or toxic (**B**; E+; n = 6) grazing beef steers. Green and red edges indicate positive and negative correlations, respectively. Select nodes of interest are highlighted by arrows and text.

Analysis of the rumen revealed one main cohort of clusters with a single cluster unattached to the main cohort in both Max-Q and E+ (Fig. 7). Additionally, in Max-Q (File S2C) and E+ (File S2D) networks, fungal OTUs had highest centrality measurements. While some fungi with high centrality measurements were present in both (e.g., *Neocallimastigaceae*), others were distinct. Fungi specific to the E+ network included *Aspergillus*, *Leptospora*, and *Fusarium* (Fig. 7; File S2D). The features with the next highest centrality measurements were bacterial OTUs and many were similar in Max-Q and E+ networks; however, *Pyramidobacter* and *Treponema* were unique to E+ (File S2C). E+ network-specific metabolic pathways included glycosphingolipid biosynthesis, metabolism of xenobiotics by cytochrome P450, and folate biosynthesis (Fig. 7).

#### Global animal integrative interactomics

Global (i.e., rumen, plasma, urine, feces; microbiota and metabolomes) xMWAS resulted in two unique networks between Max-Q and E+ steers.

The nodes with the highest centrality measurements in the E+ network were from the fecal fungal, rumen solids/liquids bacterial, and fecal bacterial features (File S2F; eigenvector centrality > 0.7; rest of nodes centrality was less than 0.3); in the Max-Q network, those nodes were fecal fungi, rumen liquids bacteria, and fecal bacteria (File S2E; centrality > 0.84; rest less than 0.3). The fecal fungal OTUs within the Max-Q and E+ networks were quite distinct, with the E+ network having two *Neocallimastigaceae Anaeromyces*, one *Aspergillus*, one *Acremonium brachypenium*, and one *Meyerozyma* OTU (File S2F). Although ruminal liquid OTUs had some overlap between the two treatments (i.e., *Lachnospiraceae*), most were distinct. The OTUs solely in the E+ network aligned to unclassified *Betaproteobacteria* and *Bacteroidetes*, one from candidates *BS11* and *LD1-PB3*, and one *Ruminococcaceae* and *Mogibacteriaceae* (File S2F). The only fecal bacterial OTUs unique to the E+ network were one *Erysipelotrichaceae* and one *Anaerolinaceae* OTU (File S2F). The rumen solid bacterial OTUs in the global E+ network were diverse (File S2F).

**Figure 8:**
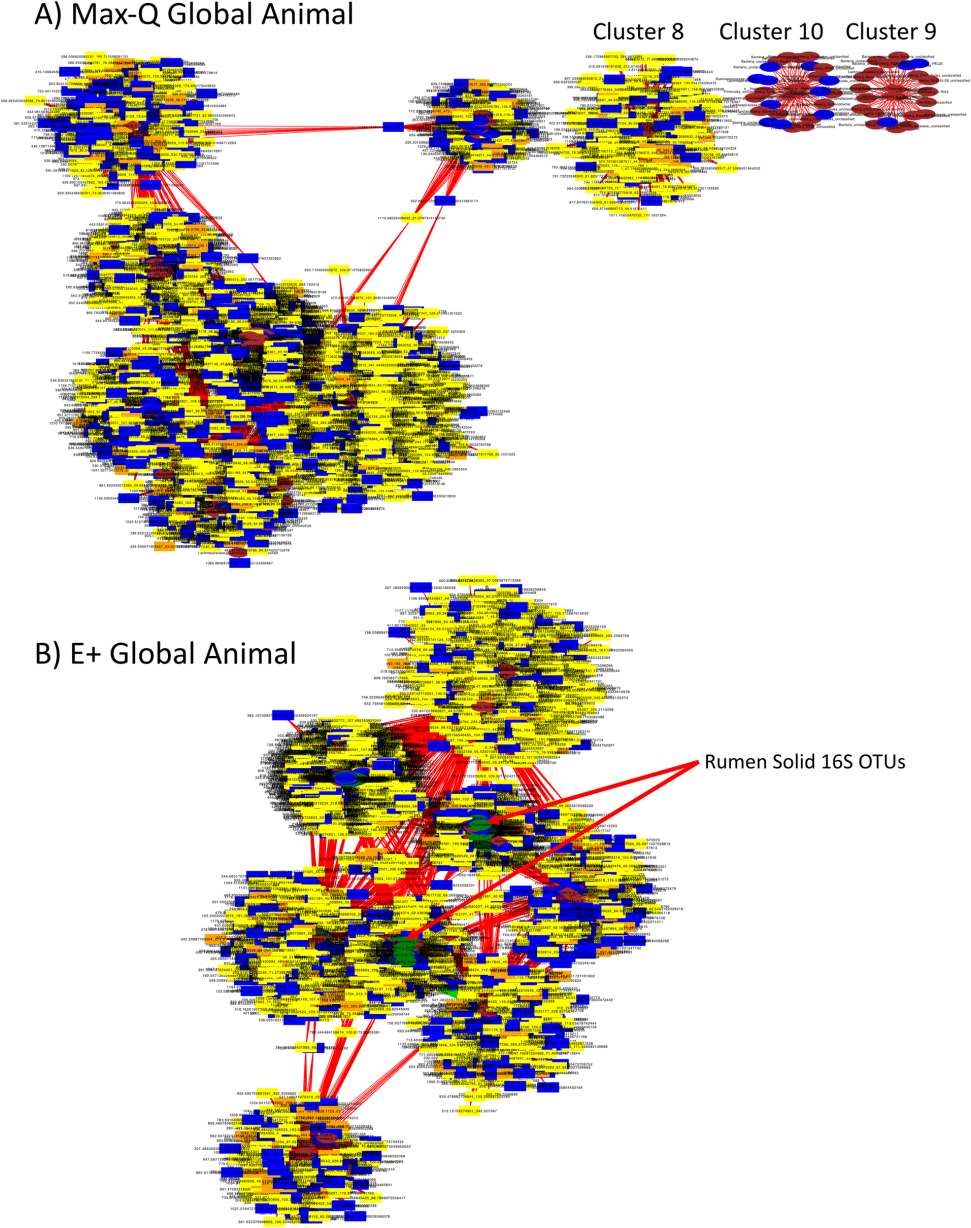
Global whole animal integrative interactomics networks of the relationships between bacterial (oval) and fungal (diamond) OTUs and metabolites (rectangle) in the rumen solid (green), rumen liquid (blue), plasma (orange), urine (yellow), and feces (brown) of either (**A**) non-toxic (Max-Q; n = 6) or (**B**) toxic (E+; n = 6) endophyte-infected tall fescue grazing beef steers. Green and red edges indicate positive and negative correlations, respectively.

Interestingly, we sought to identify metabolites with high centrality (> 0.2) that were different between the Max-Q and E+ global networks (Fig. 8). Most node metabolites were urinary metabolites, with rumen liquid and plasma metabolites making up a smaller portion. Plasma metabolites unique to the E+ network were mainly involved in fatty acid and riboflavin metabolism. The rumen metabolic features unique to the E+ networks were primarily involved in steroid biosynthesis, folate biosynthesis, and metabolism of xenobiotics by cytochrome P450. The E+-specific urinary metabolites were associated with steroid hormone biosynthesis, purine, arachidonic acid, pentose phosphate pathway, and tryptophan and tyrosine metabolism. Urinary steroid hormone biosynthesis as a pathway appeared in both networks, but the metabolic features annotated within this pathway were network-specific. The Max-Q plot had three stand-alone clusters apart from the main network structure (Fig. 8A), which was not seen in the E+ network (Fig. 8B). Full node tables for the fescue plant, rumen, and global xMWAS analyses can be found in File S2.

#### Targeted animal integrative interactomics analysis

**Figure 9:**
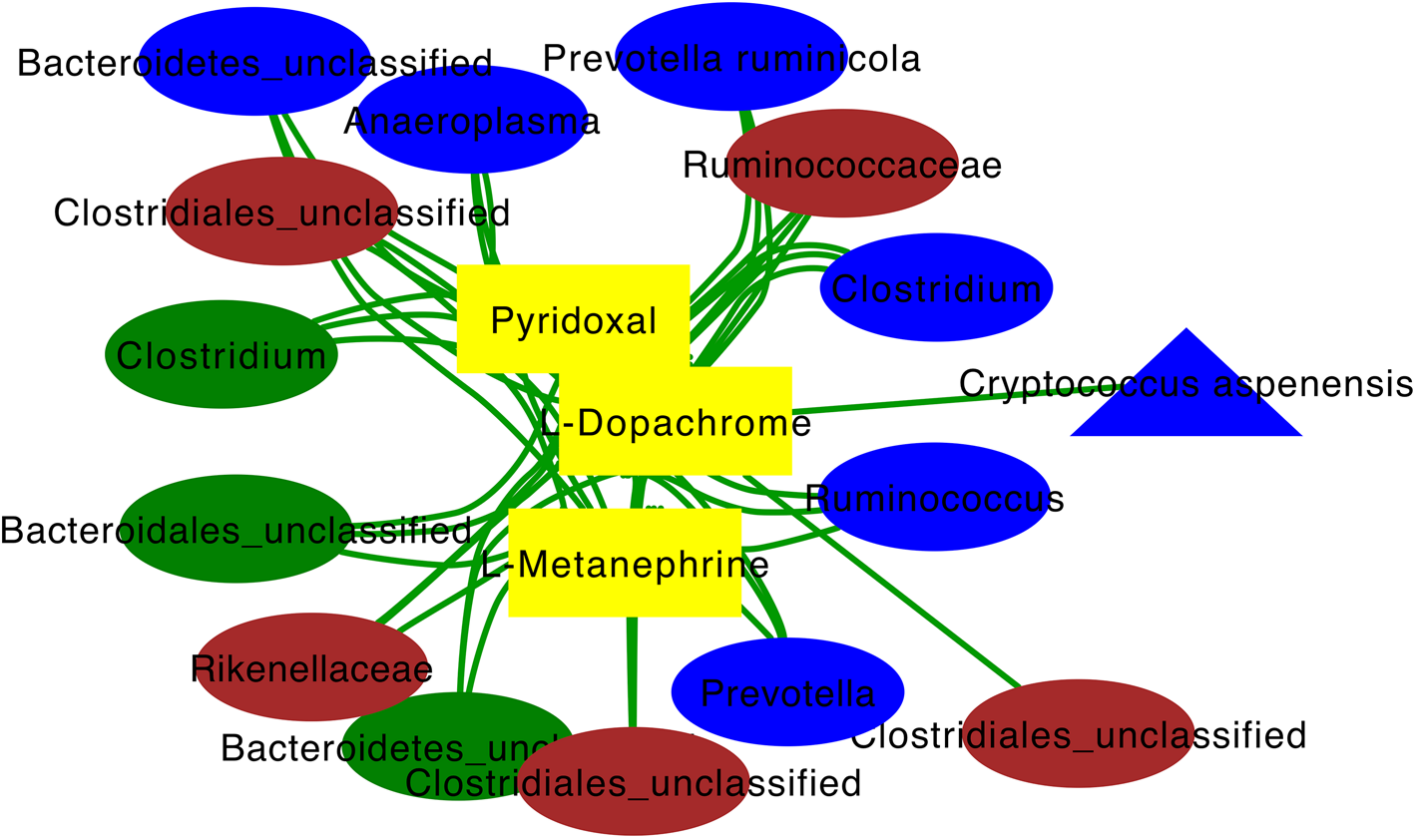
Targeted whole animal integrative interactomics networks of the relationships between bacterial (oval) and fungal (triangle) OTUs and metabolites (rectangle) in the rumen solid (green), rumen liquid (blue), urine (yellow), and feces (brown) of steers on toxic (E+; n = 6) endophyte-infected tall fescue. Metabolic pathways targeted in this analysis include tryptophan, tyrosine, Vitamin B6, steroid hormone, and bile acid metabolism. Green edges indicate positive correlations.

Targeted analysis assessed the multi-compartment relationship between metabolites and microbes that were significantly affected by E+ and present solely in a targeted E+ integrome network. One resultant cluster specific to E+ was centered on three urinary metabolic features that were highly associated (|r| > 0.7) with bacterial and fungal OTUs from every animal biological matrix (Fig. 9). The three metabolites central to this network are l-metanephrine, l-dopachrome, and pyridoxal, which are involved in tyrosine and Vitamin B6 metabolism, respectively (Fig. 9). OTUs associated with all three metabolites in the network include: *Anaeroplasma*, *Prevotella ruminicola*, *Clostridium*, *Ruminococcus*, and *Prevotella* (rumen liquids), *Clostridium* (rumen solids), *Ruminococcaceae*, *Rikenellaceae* (feces), other unclassified OTUs in all matrices (Fig. 9). Notably, one unclassified fecal bacterial OTU and one rumen liquid *Cryptococcus aspenensis* OTU were associated solely with urinary L-dopachrome in the targeted integrome network (Fig. 9).

## Discussion

Herein, is the first fescue toxicosis analysis using an integrative multi-‘omics approach that includes both the plant and animal. *E. coenophiala* infection and exposure significantly altered both the plant and animal multi-compartment microbiota and metabolomes, and led to unique integrome structure. Further, while there is little overlap between the plant and animal microbiota, some E+-associated metabolic changes were common to all biological matrices.

*E. coenophiala* infection altered the tall fescue phyllosphere microbiota, including increases in numerous plant-specific bacteria and fungi, g_*Epichloë* included. Plant microbiota is influenced by plant/environmental factors, including by endophytes such as *E. coenophiala*^18–20^. Most bacteria/fungi affected by E+ in the plant and animal were distinct, but it is significant that the *Epichloë* genus was increased in the E+ rumen liquids. While it is unlikely that aerobic microbes, fungi included, are able to thrive in the anaerobic ruminal environment, one study has found that anaerobic fungi within the GI tract of cattle have one life cycle stage that provides increased aerobic tolerance^39^, suggesting some tolerance flexibility. Considering this and reports of aerobic fungi being viable in bovine feces^40^, one explanation for the number of fungal OTUs that overlapped between all biological matrices is that the complex life cycle and sporulation of fungi could allow them to persist in adverse environments^39,40^.

Across the grazing trial, E+ exposure reduced most ruminal fungal taxa in the solid and liquid fractions. Fungi digest cellulose in the rumen; mycelium penetration of feed particles breaks fibers apart, increasing surface area for better degradation^41^. Their ability to degrade fibrous particles of feedstuffs is an important part of ruminant nutrient extraction^41^. Increased rumen fill, unexplained by increased dry matter intake, has been reported in FT^6,42–44^ and might reflect decreased ruminal passage rates. In this study, we noticed, but did not quantify, that E+ steers had greater rumen solid contents fraction. Considering the role ruminal fungi play in feed degradation, the relationship between fungal shifts in response to E+ exposure and alterations in rumen fill/passage rates should be evaluated, especially considering that specific fungal microbiota may provide tolerance to E+ exposure^11^.

While plant–animal carryover effects were limited to the microbiota, several metabolic pathways were affected by E+ in both the plant and animal. Some (e.g., tryptophan) pathways align with our earlier E+ grazing studies^12–14^, but we also identified other important pathways, such as Vitamin B6 metabolism, as significantly perturbed in all biological matrices. We previously reported altered urinary Vitamin B6 metabolism in fescue toxicosis^14^. Here, we found it altered in all biological matrices. Multiple forms of Vitamin B6 exist^45^ and it is an essential vitamin in humans and animals^46^. While plants synthesize it de novo, animals must obtain it through diet^47,48^. Alterations in Vitamin B6 metabolism in the plant and rumen could perturb downstream amino acid metabolism through microbial means^49^. Also, multiple enzymes in tryptophan metabolic pathways (e.g., kynureninase) use Vitamin B6 as an essential co-factor and its reduction decreases tryptophan metabolites in mice^50,51^. Notably, l-kynurenine was an E+-specific metabolite we found overlapping between all biological matrices (plant and animal). Vitamin B6 is also a co-factor for transaminases^52^ that are induced by glucocorticoids following stress, indicating possible widespread effects of Vitamin B6 alterations. Overall, plant and animal alterations in Vitamin B6 metabolism could influence subsequent amino acid metabolism that is consistently altered in E+ grazing studies^13,14^. Folate (Vitamin B9) biosynthesis was a metabolic pathway that appeared only in the E+ rumen and global integrative networks. Previously, it was demonstrated that thiamin (Vitamin B1) supplementation provided benefits to E+ grazing steers^53^. Thus, the relationship between vitamins in the B family with FT pathophysiology and, perhaps, broader vitamin supplementation/monitoring as a FT therapeutic is worthy of further investigation.

Interestingly, this study putatively identified ergovaline, the most prevalent toxic fescue ergopeptine^7,54^, via untargeted metabolomics. This is novel and confirming this metabolic feature by targeted means is important. The pattern of putative ergovaline detection aligns with what would be expected from the literature. No ergovaline was detected in biological matrices where he non-toxic Max-Q endophyte was used, which is expected since this endophyte was created to not produce ergot alkaloids while providing other agronomic benefits^55–57^. In E+, putative ergovaline was found in the fescue plant and rumen. In the plant, one-third of E+ samples had this *m/z*. Whether this is due to the untargeted metabolomics-based, non-ergot alkaloid specific^58^ extraction method, varied level of endophyte infection, small sample size, or other factors, is unknown. Given the small variability of stem and leaf ergovaline levels measured by targeted analysis^59^, small sample amount in combination with the broad, untargeted metabolomics extraction method is likely responsible for the non-uniform detection.

In the rumen of E+ steers, putative ergovaline was undetected before pasture placement, with levels increasing until peaking at Day 14. Urinary ergot alkaloids in this study indicate these steers were not recently exposed to toxic tall fescue pastures, so it was not expected to detect ergovaline on Day 0. The pattern of ergovaline in the rumen follows a similar pattern to total urinary ergot alkaloids, a sensitive biomarker of ergot alkaloid exposure^24^, until Day 14 of the grazing trial. Nominal decrease in ruminal ergovaline on Day 28 of the grazing trial could have multiple origins. We have found that urinary ergot alkaloid levels plateau or decrease and differences between the E+ and Max-Q microbiota become stable after 14 days of grazing E+ fescue in the fall, spring, and early summer^12–14^. This plateau, including herein, across seasons may indicate an adaptive response to E+ after 14 days on pasture and/or that steady state metabolism has been reached. If the animal microbiota shifted and/or cytochrome P450 levels increased^60,61^ to adapt to ergovaline and other ergot alkaloids, accelerated metabolism and/or biotransformation into less toxic metabolites would take place and result in decreased ruminal ergovaline towards the end of the study as observed here.

Targeted plant and rumen network analysis revealed most OTUs associated with the *E. (coenophiala*) OTU were distinct between the plant and animal. The bacterial family *Lachnospiraceae*, a family commonly affected by E+ in the grazing animal^12–14^, and the *Orpinomyces* genus were the only microbes that had OTUs significantly associated with *E. (coenophiala*) in both matrices. Notably, none of the *Lachnospiraceae* or *Orpinomyces* OTUs in the plant and the animal overlapped, indicating that sub-genus differences exist between what is present in the plant and the animal. The only fungus that was associated with *E. (coenophiala*) and was increased in the plant was one *Leptospora sp* OTU. A member of the *Phaeosphaeriaceae* family, which contains economically costly plant pathogens, some *Leptospora sp* relatives, have been identified as endophytes in monocotyledons plants^62^, like tall fescue. The genera *Sphingomonas* and *Methylobacterium* were associated with *E. (coenophiala*), but were decreased in E+ tall fescue. The relationship between them and *E. (coenophiala*) is unclear, but it seems possible that they could be competing for resources with *Epichloë* and/or be influenced by *Epichloë* secondary metabolites.

Within the rumen, most correlating OTUs were bacteria, but some notable fungi associated with *E. (coenophiala*) OTU too. The *Neocallimastigaceae* family, namely the *Orpinomyces* genus, had the majority of fungal *Epichloë*-associated fungal OTUs. Notably, two *Orpinomyces* OTUs were correlated with *Epichloë* in both the rumen solids and rumen liquids, one positively and one negatively. Of the 15 rumen liquid *Prevotella* OTUs, 10 were negatively correlated and 5 were positively correlated; of the 13 rumen solid OTUs, 3 were negatively correlated and 10 were positively correlated. Overall, for most taxa with multiple correlations, there was a mix of positively and negatively correlated OTUs, indicating that sub-genus targeted analysis of these fungi/bacteria might help understanding the complex rumen microbiota relationship in FT. Such work, in a different context, has outlined how different strains of active dry yeast influence ruminal acidosis and methane production^63^ and found strain-specific carbohydrate-utilization patterns in ruminal bacteria^64^. These data highlight the feasibility of a sub-genus, targeted microbiota analyses in FT context.

Targeted ergovaline network analysis revealed highly interconnected network between rumen metabolites. The majority of the metabolites in this network were involved in steroid hormone biosynthesis, tryptophan, tyrosine, and Vitamin B6 metabolism. This is interesting considering these metabolites are components of metabolic pathways most significantly affected by E+ grazing. Previously, we found that E+ altered tryptophan and tyrosine metabolism^13,14^. So, while putative ergovaline did not have the highest centrality in the full network, the metabolic pathways it was associated with were the same ones identified by broader metabolomics methods. In the focused networks, the only unique affected pathways were steroid hormone and primary bile acid biosynthesis and ergovaline had the highest centrality of all metabolic features. It has been previously shown that ergot alkaloids can influence systemic hormonal homeostasis^65^, but current data indicate ergovaline and E+ tall fescue grazing may begin to induce hormonal imbalances presystemically, i.e., in the rumen; however, this will require further investigation.

Integrative analysis revealed the general structures of the global Max-Q and E+ networks were similar (i.e., microbial nodes as anchors with peripherally associated metabolites), but the constituents of the networks were distinct. Notably, in both networks, fecal fungal OTUs had the highest centrality measurements, but the fungal classifications were mostly network-specific. One of the E+-unique genera was fecal *Neocallimastigaceae Anaeromyces*. Although *Anaeromyces* was not a genus reported, it was previously found that the *Neocallimastigaceae* family was increased in steers with greater tolerance to E+ exposure, indicating this family may be important in the structure of the E+ integrome and play a modulatory role in the severity of FT^11^. Considering that E+ reduced the abundance of most fungal taxa, yet ruminal solid fungal OTUs appear only in the E+ integrome, further exploration of the specific influence of E+ on ruminal fungi homeostasis is warranted.

Previously, urinary ergot alkaloids have been proposed as a sensitive biomarker of exposure^12–14,24,25^ and, potentially, a biomarker of effect^24^ for E+ in beef cattle. Although these provide great utility for producers and scientists alike, additional biomarkers that encapsulate the molecular mechanisms of E+-induced decreased weight gains may be therapeutically valuable. In our search for subsequent/supplemental biomarkers, we identified a ruminal *Epichloë* OTU only in E+ animals that was most abundant after 14 days of grazing. As this E+ specific OTU did not track well with pasture alkaloids, urinary ergot alkaloids, or weight gains in E+ steers, its identification is interesting, likely consequential, but not an ideal biomarker as its presence might be related to endophyte breakdown together with the plant material in the rumen.

Akin to what we found for plasma/urinary metabolites having utility as a biomarker of a decreased productivity-associated hindgut microbiota^12^, targeted global integrative analyses performed herein revealed three urinary metabolic features (l-metanephrine, l-dopachrome, and pyridoxal) positively associated with the E+ microbiota in multiple animal matrices. All three were urinary metabolites, not plasma or rumen liquid, indicating urine can be discriminatory between Max-Q and E+ steers and ideal for easy-to-access biomarkers of FT. Urinary metanephrines have shown equal utility as plasma metanephrines as a diagnostic biomarker of pheochromocytoma^66^ and urinary dopachrome tautomerase protein has been suggested as a potentially sensitive biomarker of drug-induced liver injury^67^. We reported that several urinary catecholamines in E+ steers after 28 days on pasture are altered^13^. This, together with the current results, suggests pathways associated with urinary catecholamines are consistently perturbed in FT. The positive association of metanephrine and dopachrome with microbiota from multiple compartments hints that these urinary metabolites may be useful biomarkers for FT from a therapeutics perspective. Urinary pyridoxal was the other metabolite appearing in this focused network, which is notable, considering Vitamin B6 metabolism was perturbed in all plant and animal matrices tested. Finally, these data provide foundational evidence that show the previous perturbations we have identified^12–14^ are snapshots of systemic perturbations that occur in E+ grazing steers; understanding the multi-level, multi-compartment, integrome will provide more actionable insights. Although the relationship between urinary l-dopachrome and *Cryptococcus aspenensis* in FT context is unclear, it is interesting that l-dopachrome was the only feature associated with this fungal OTU.

In this novel study, effects of *E. coenophiala* infection on the plant and animal microbiota, metabolome, and the multi-compartment, multi-‘omics integration are presented. The data suggest the majority of the microbiota profiles, and the effects of E+, are distinct between the plant and the animal, but effects of E+ on the plant and multi-compartment animal metabolome shared some similarities. Herein, is the first overview of the complex interactions between the bovine multi-compartment microbiota, both bacterial and fungal, and metabolome; these relationships are endophyte-specific. We found that only the E+ integrome had rumen solid OTUs, indicating these may be an important microbial point-of-origin for E+ pathophysiology. Overall, these data align with our previous work showing E+ disrupts plasma and urine metabolic and fecal microbiota homeostasis^12–14^. Additionally, our current finding that E+ begins altering the microbiota/metabolome *in planta* and in the rumen of toxic fescue grazing steers and these changes associate with plasma/urine metabolic and fecal microbiota changes is novel; it suggests a complex, systemic pathophysiological response in FT. Future therapeutic- or management-based intervention strategies, as well as detailed evaluation of adaptive vs pathophysiological responses to E+ and in the context of other complex diseases, should take advantage of such integrome-based integrative analyses.

## Supplementary Information

Supplementary Information 1.

Supplementary Information 2.

Supplementary Information 3.

Supplementary Information 4.

Supplementary Information 5.

Supplementary Information 6.

Supplementary Information 7.

Supplementary Information 8.

Supplementary Information 9.
