# Supplementary material for: Integrative interactomics applied to bovine fescue toxicosis
Source: Sci Rep. 2022 Mar 22;12:4899. doi: 10.1038/s41598-022-08540-2 (PMC8941056; doi:10.1038/s41598-022-08540-2)
Supplement: Supplementary file 1 — Supplementary Information 1. [file 41598_2022_8540_MOESM1_ESM.docx]

**Figure S1. (A)** Urinary ergot alkaloids (ng/mg creatinine) and **(B)** average daily gain (ADG; kg/day) and cumulative gain (kg) in Angus steers that grazed either non-toxic endophyte-infected (Max-Q; n=6) or toxic endophyte-infected (E+; n=6) tall fescue for a 28-day grazing trial. (*) P < 0.05, (***) P < 0.001.

**Figure S2.** Changes in diversity (Simpson’s diversity index) and richness (Chao1 richness) metrics in Angus steers and tall fescue grazing or being infected with either a non-toxic endophyte (Max-Q; n= 6) or a toxic endophyte (E+; n = 6) before placement on pastures (Day 0) and for 1, 2, 14, and 28 days (bovine samples) or Day 0, 14, and 28 (fescue samples). Data are presented as mean with standard deviation as error bars. White and black bars indicate Max-Q and E+ tall fescue or grazing steer treatment, respectively.

**Figure S3.** Linear discriminant analysis (LDA) effect size (LEfSe; Kruskall-Wallis [P < 0.05]; Pairwise Wilcoxon [P < 0.05]; logarithmic LDA score > 2.0) of tall fescue cultivar **(A)** bacterial and **(B)** fungal microbiota across a 28-day grazing trial where green and red indicate significant increases in the non-toxic (Max-Q) and toxic (E+) tall fescue cultivars, respectively. Taxonomic rank labels are provided before bacterial names: “p_; c_; o_; f_; g_” indicate phylum, class, order, family, and genus, respectively. Letters and numbers within the cladogram refer to respective bacterial or fungal names located in the keys to the right of each cladogram. Select taxa of interest have been highlighted by boxes and arrows point to position within cladogram.

**Figure S4.** Linear discriminant analysis (LDA) effect size (LEfSe; Kruskall-Wallis [P < 0.05]; Pairwise Wilcoxon [P < 0.05]; logarithmic LDA score > 2.0) of the rumen solid **(A)** bacterial and **(B)** fungal and rumen liquid **(C)** bacterial and **(D)** fungal microbiota of Angus steers across a 28-day grazing trial after placement on either a non-toxic (Max-Q; n = 6) or toxic (E+; n = 6) endophyte-infected tall fescue. Blue, green and red shading indicates greater abundance in Pre (before pasture placement), Max-Q, or E+ steers, respectively. Taxonomic rank labels are provided before bacterial names: “p_; c_; o_; f_; g_” indicate phylum, class, order, family, and genus, respectively. Letters and numbers within the cladogram refer to respective bacterial or fungal names located in the keys to the right of the cladogram. Select taxa of interest have been highlighted by arrows and text.

**Figure S5.** Linear discriminant analysis (LDA) effect size (LEfSe; Kruskall-Wallis [P < 0.05]; Pairwise Wilcoxon [P < 0.05]; logarithmic LDA score > 2.0) of the **(A)** bacterial and **(B)** fungal fecal microbiota of Angus steers across a 28-day grazing trial after placement on either a non-toxic (Max-Q; n = 6) or toxic (E+; n = 6) endophyte-infected tall fescue. Green and red shading indicates greater abundance in Max-Q or E+ steers, respectively. Taxonomic rank labels are provided before bacterial names: “p_; c_; o_; f_; g_” indicate phylum, class, order, family, and genus, respectively. Letters and numbers within the cladogram refer to respective bacterial or fungal names located in the keys to the right of the cladogram. Select taxa of interest have been highlighted by boxes and arrows point to position within cladogram.

**Figure S6.** Linear discriminant analysis (LDA) effect size (LEfSe; Kruskall-Wallis [P < 0.05]; Pairwise Wilcoxon [P < 0.05]; logarithmic LDA score > 2.0) of the **(A)** bacterial and **(B)** fungal fecal microbiota of Angus steers across a 28-day grazing trial after placement on either a non-toxic (Max-Q; n = 6) or toxic (E+; n = 6) endophyte-infected tall fescue. Blue, green and red shading indicates greater abundance in Pre (before pasture placement), Max-Q, or E+ steers, respectively. Taxonomic rank labels are provided before bacterial names: “p_; c_; o_; f_; g_” indicate phylum, class, order, family, and genus, respectively. Letters and numbers within the cladogram refer to respective bacterial or fungal names located in the keys to the right of the cladogram. Select taxa of interest have been highlighted by arrows and text.

**Figure S7.** Targeted correlation-based network analysis including rumen metabolic features significantly correlated with ergovaline in the overall network of toxic tall fescue grazing steers (E+; |r| > 0.6; P < 0.05). Green and red indicate positive and negative correlations, respectively.

**File S1. and S2.** Containing overlapping OTUs names and biological matrices where present (S5.1.) and xMWAS tables with raw name, cluster, centrality measurement, annotation, shape, and color can be found in the following public folder:

https://drive.google.com/drive/folders/1jYOyZi1Fy4f0UCGlncPs81kDdHZxQyA6?usp=sharing
