# Supplementary material for: Integrative interactomics applied to bovine fescue toxicosis
Source: Sci Rep. 2022 Mar 22;12:4899. doi: 10.1038/s41598-022-08540-2 (PMC8941056; doi:10.1038/s41598-022-08540-2)
Supplement: Supplementary file 2 — Supplementary Information 2. [file 41598_2022_8540_MOESM2_ESM.docx]

**Supplemental Table 1.** Effects on the overall microbial profile (PERMANOVA) using abundance profiles (Bray-Curtis) or presence absence (Jaccard) and diversity in richness for the bacterial (16S) and fungal (ITS2) microbiota based on fescue treatment (Max-Q [non-toxic] vs E+ [toxic]), time spent grazing, and the treatment by time interaction in fescue cultivar, rumen solids, rumen liquids, and fecal matter. “Inc”, “dec”, “mix” indicate significant increase, decrease or mixed effects, respectively, by E+ and/or time. P values equate to: (---) > 0.1; (^) < 0.1; (*) < 0.05; (**) < 0.01; (***) < 0.001.

| **Biological Matrix** | **PERMANOVA**  **(Bray-Curtis)**  **16S ITS2** | **PERMANOVA**  **(Jaccard)**  **16S ITS2** | **Simpson’s Diversity**  **16S ITS2** | **Chao1 Richness**  **16S ITS2** |
| --- | --- | --- | --- | --- |
| Fescue cultivar  Treatment  Time  Treatment:Time | * ^  ** ***  *** ^ | ** ^  *** ***  ** ^ | * (inc) ^(inc)  ^(mix) ***(mix)  --- --- | --- ---  --- **(mix)  --- --- |
| Rumen Solids  Treatment  Time  Treatment:Time | *** ***  *** ***  --- * | *** ***  *** ***  --- * | --- ***(inc)  --- ---  --- --- | ***(inc) ---  ***(dec) ---  --- --- |
| Rumen Liquids  Treatment  Time  Treatment:Time | *** ***  *** ***  --- * | *** ***  *** ***  --- * | **(dec) ---  ***(inc) **(inc)  --- --- | ***(inc) ---  **(dec) ***  --- --- |
| Feces  Treatment  Time  Treatment:Time | *** **  *** **  --- --- | *** **  *** **  --- --- | --- ---  --- *(mix)  --- --- | ***(mix) ^(mix)  **(mix) ---  --- * |
