# Supplementary figures and images for: Integrative interactomics applied to bovine fescue toxicosis
Source: Sci Rep. 2022 Mar 22;12:4899. doi: 10.1038/s41598-022-08540-2 (PMC8941056; doi:10.1038/s41598-022-08540-2)

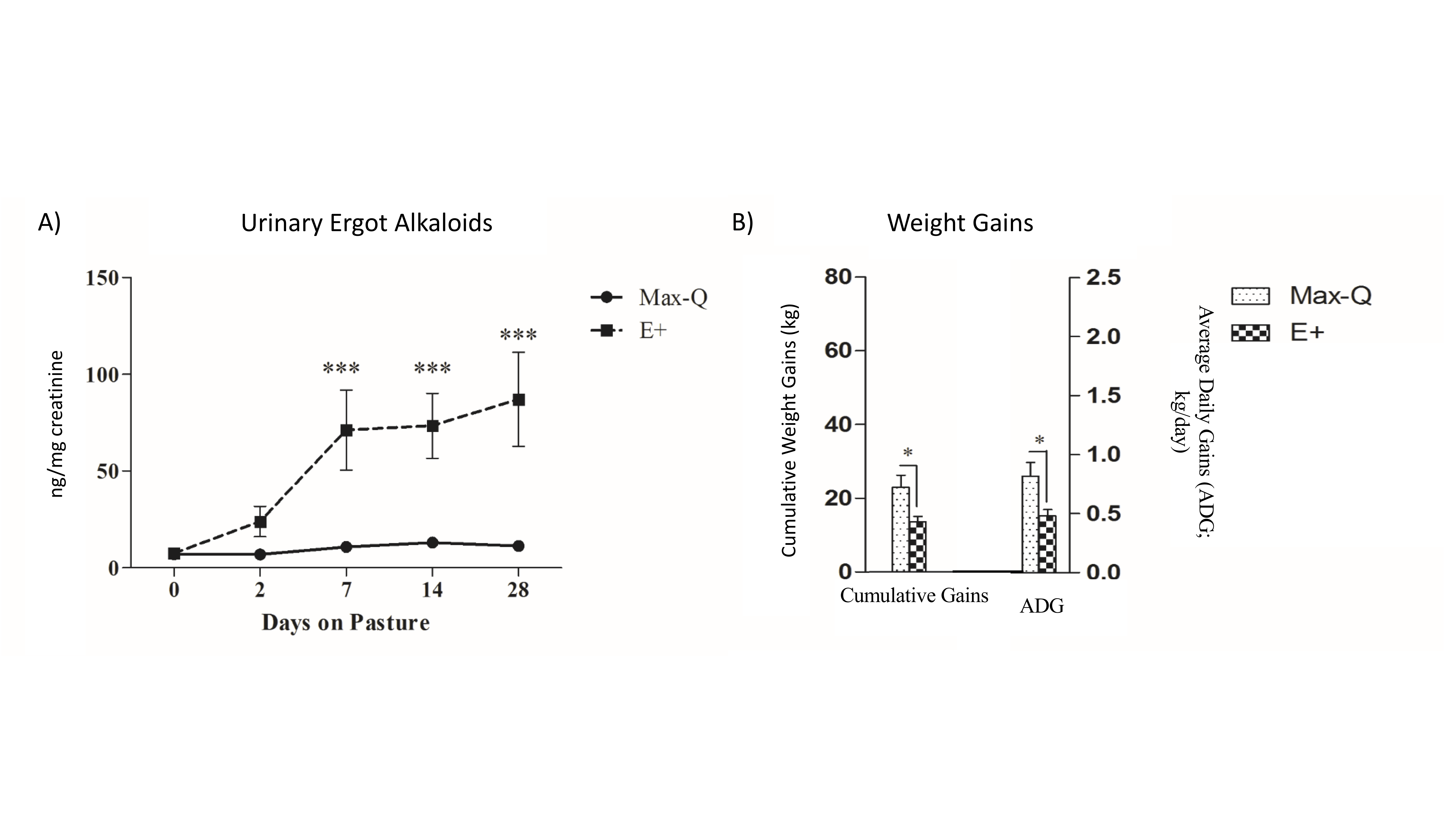

Supplement: Supplementary file 3 — Supplementary Information 3. [file 41598_2022_8540_MOESM3_ESM.tiff]

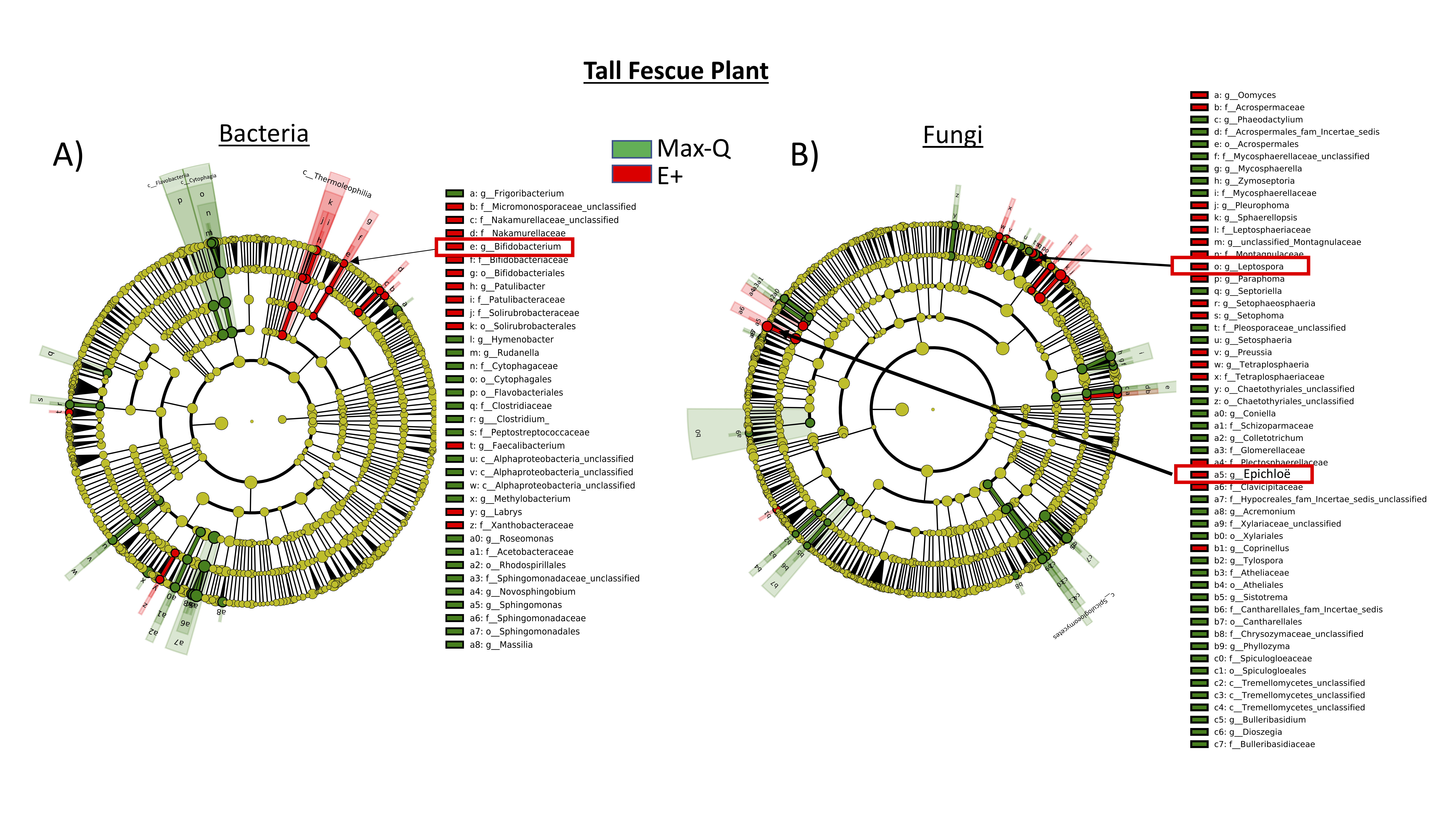

Supplement: Supplementary file 5 — Supplementary Information 5. [file 41598_2022_8540_MOESM5_ESM.tiff]

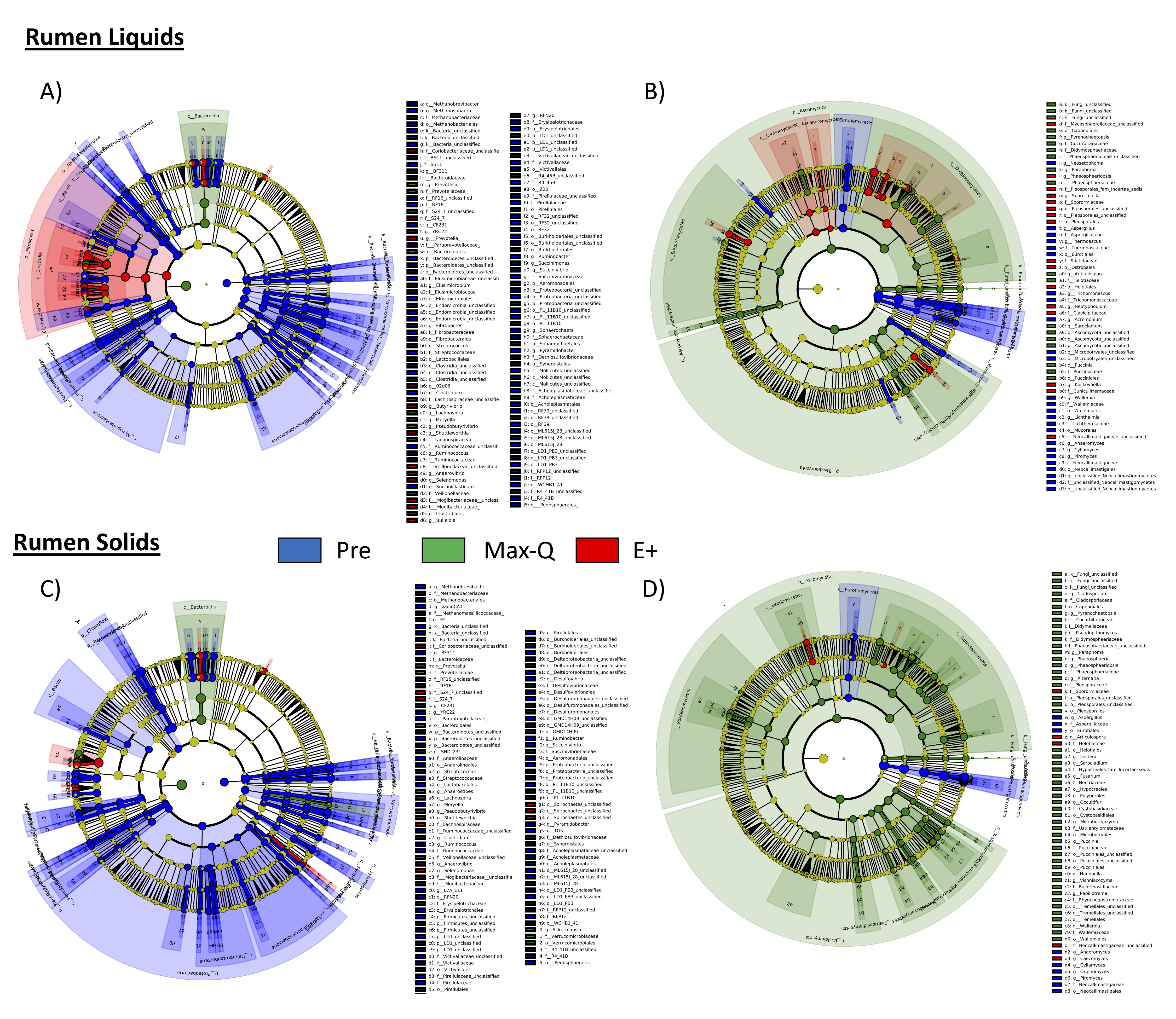

Supplement: Supplementary file 6 — Supplementary Information 6. [file 41598_2022_8540_MOESM6_ESM.tiff]

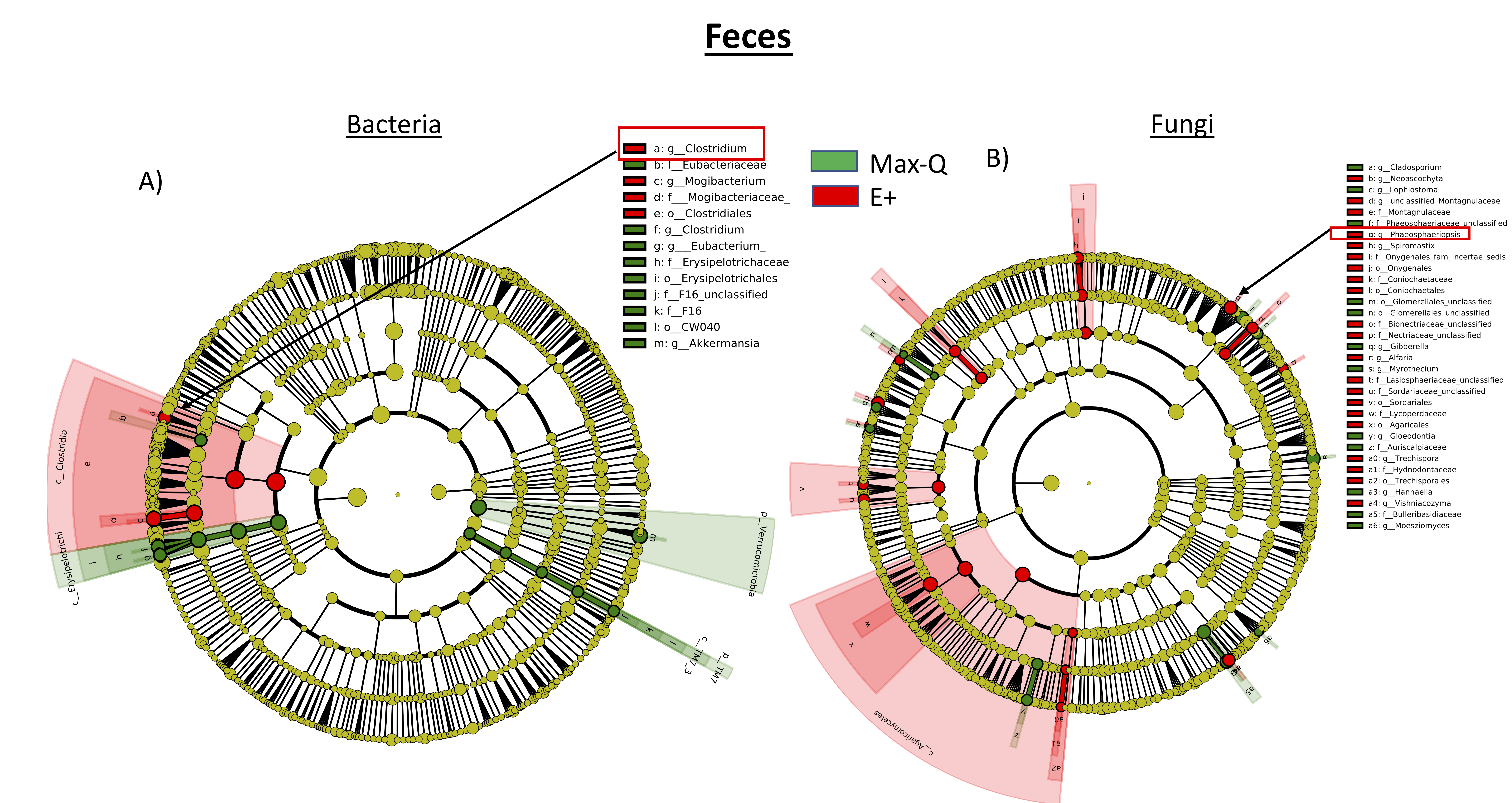

Supplement: Supplementary file 7 — Supplementary Information 7. [file 41598_2022_8540_MOESM7_ESM.tif]

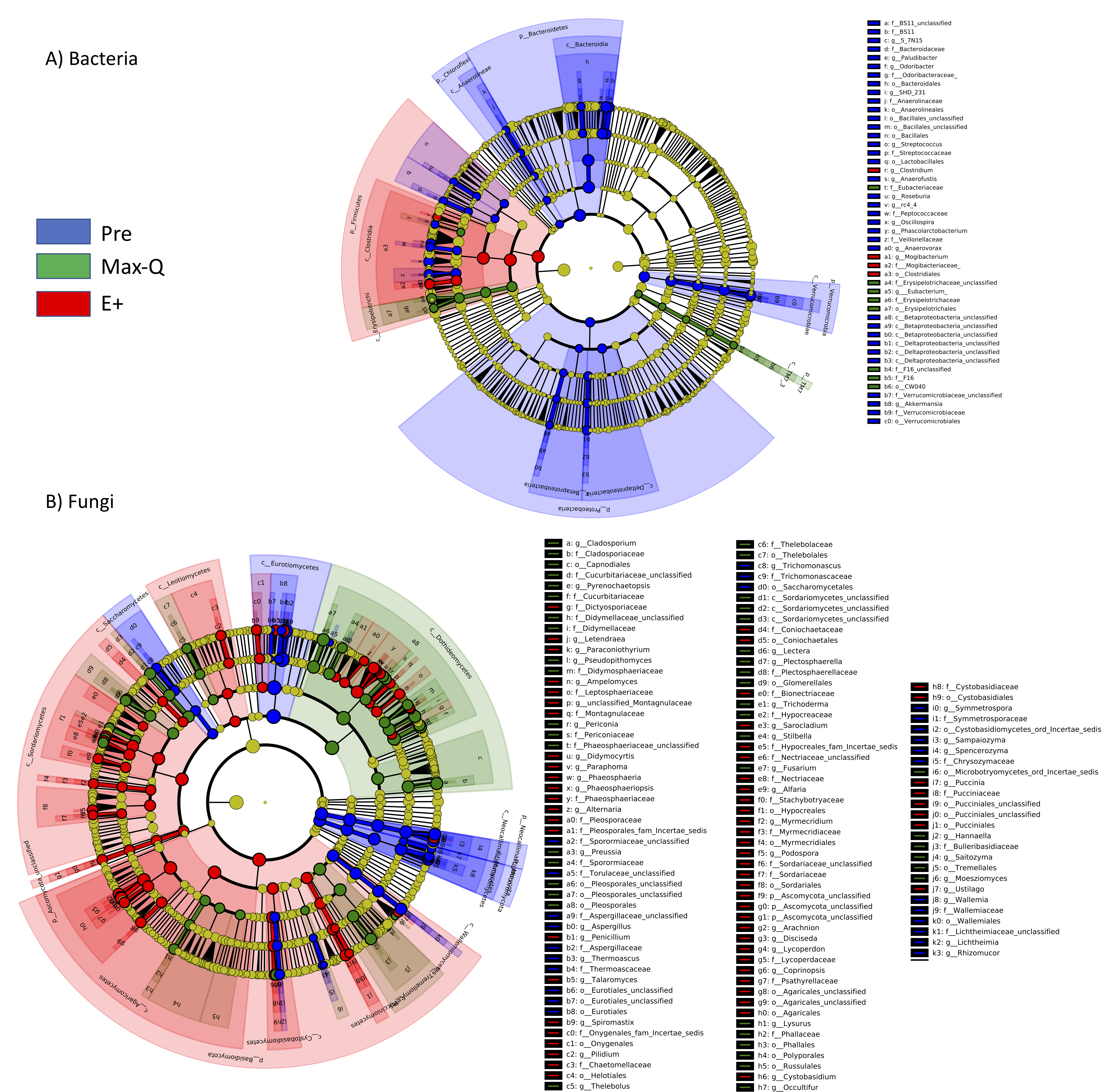

Supplement: Supplementary file 8 — Supplementary Information 8. [file 41598_2022_8540_MOESM8_ESM.tiff]

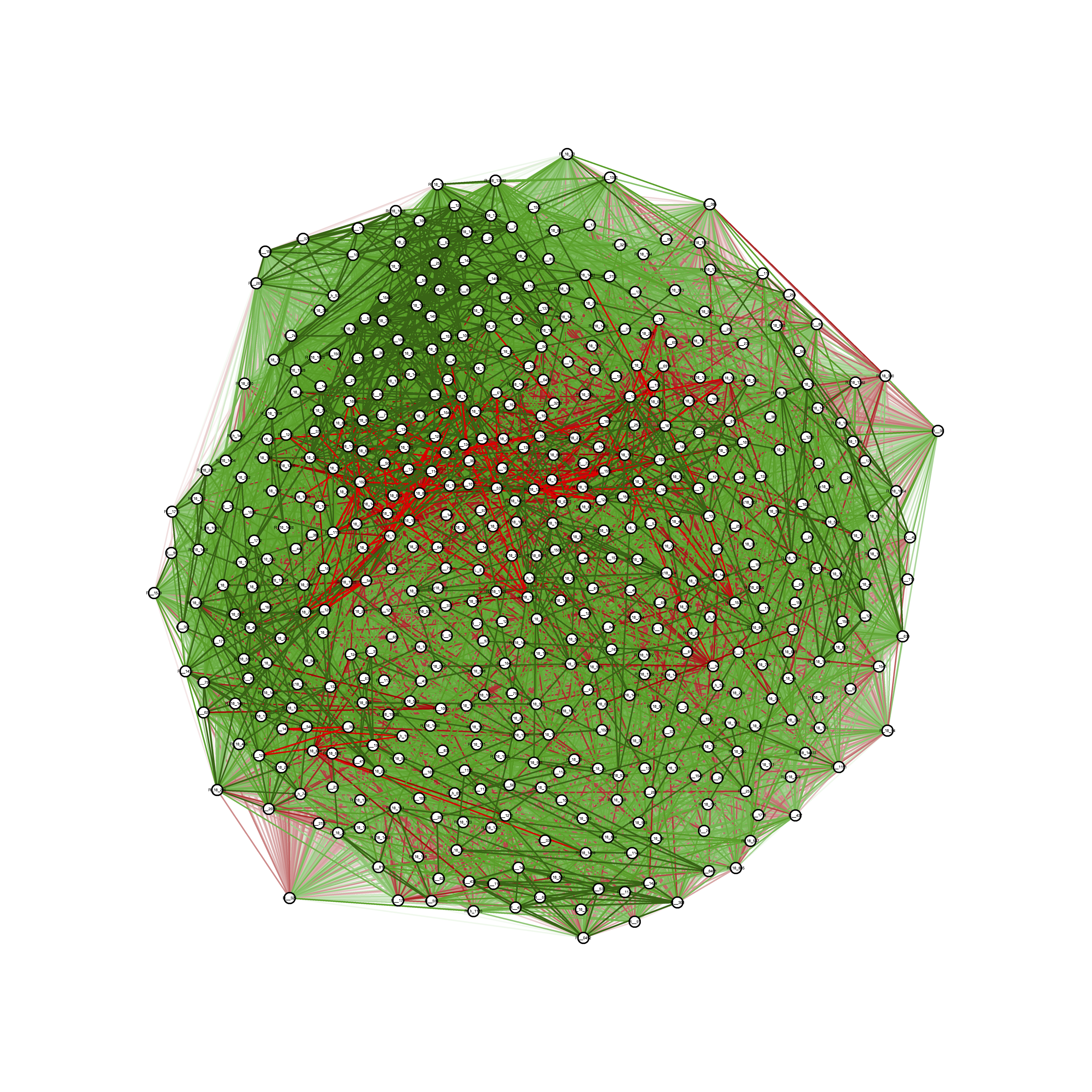

Supplement: Supplementary file 9 — Supplementary Information 9. [file 41598_2022_8540_MOESM9_ESM.tiff]
